# The efficacy of angiogenesis inhibitors combined with chemotherapy in advanced breast cancer: a systematic review and meta-analysis

**DOI:** 10.3389/fonc.2026.1820120

**Published:** 2026-06-05

**Authors:** Jiangzhuo Wu, Hanbing Li, Ling Wei, Xiao Yan, Jiang Fang, Lin Peng, Xiaobo Zhao

**Affiliations:** 1 Department of Thyroid and Breast Surgery, Affiliated Hospital of North Sichuan Medical College, Nanchong, Sichuan, China; 2 National Clinical Key Specialty (General Surgery)/National Clinical Research Center for Digestive Diseases/Sichuan Clinical Research Center For Digestive Diseases, Nanchong, Sichuan, China

**Keywords:** angiogenesis inhibitors, breast cancer, chemotherapy, meta-analysis, survival

## Abstract

**Clinical trial registration:**

Globally, breast cancer is the most common malignancy in women. Despite treatment advances, 20–30% of early-stage patients progress to advanced disease, which remains largely incurable with a 5-year survival rate of only ~20%. Chemotherapy, the current mainstay, has reached a therapeutic plateau, with limited efficacy and potential pro-metastatic effects. Anti-angiogenic agents targeting VEGF/VEGFR2 (e.g., bevacizumab, TKIs) are used clinically, but their benefit in advanced breast cancer is controversial: progression-free survival (PFS) gains are inconsistent, overall survival (OS) benefits are unclear, and resistance with class-specific toxicities (e.g., hypertension) is common. Furthermore, comparative efficacy across drug classes and optimal patient selection remain undefined. These unresolved issues highlight the urgent need for a comprehensive synthesis to guide clinical decisions and future research.

**Methods:**

Systematic search of PubMed/Web of Science (up to July 16, 2025) identified 29 phase II/III RCTs (N = 8,480) comparing angiogenesis inhibitors + chemotherapy vs. chemotherapy alone (± placebo) in advanced breast cancer. Outcomes included PFS, OS, objective response rate (ORR), clinical benefit rate (CBR), disease control rate (DCR), and safety. Two independent reviewers performed screening, extraction, and quality assessment. Pooled HRs (95% CI) for PFS/OS; ORs for binary outcomes. Random-effects model used if I² ≥ 50%; prespecified subgroup/sensitivity analyses explored heterogeneity.

**Results:**

This meta-analysis (29 RCTs, N = 11,068) showed that adding angiogenesis inhibitors in advanced breast cancer significantly improved PFS (HR 0.75), ORR, CBR, and DCR (all P<0.001), but not OS (HR 0.95, P = 0.171). PFS benefit varied by subtype: mAbs (e.g., bevacizumab) outperformed TKIs in TNBC (HR 0.59 vs. 0.75); TKIs trended better in HR+ disease (HR 0.67). Benefit was consistent across metastasis patterns but greater in patients without bone metastasis. Safety risks increased significantly, including hypertension (OR 4.59), thrombocytopenia (OR 4.54), proteinuria (OR 2.38), hand-foot syndrome (OR 2.14), and diarrhea (OR 1.97).

**Conclusions:**

This meta-analysis (29 RCTs) finds that adding angiogenesis inhibitors to chemotherapy significantly improves PFS and response in advanced breast cancer—especially in TNBC with mAbs and HR+ disease with TKIs—but not OS. Benefit is independent of visceral metastasis but reduced in bone metastases. Increased toxicities (hypertension, proteinuria, hand-foot syndrome, diarrhea) warrant proactive management.

## Introduction

Breast cancer represents the most prevalent malignant tumor among women globally, with an annual incidence exceeding 2 million cases, and stands as one of the primary causes of cancer-related mortality in the female population (1). Approximately 20% to 30% of patients diagnosed with early-stage breast cancer will ultimately progress to advanced (recurrent or metastatic) disease (2).The prognosis for advanced breast cancer remains poor, with a five-year survival rate of only about 20%, a stark contrast to the rate exceeding 90% observed in early-stage cases. Critically, advanced breast cancer is currently considered incurable. Consequently, the therapeutic paradigm has shifted from seeking a cure to focusing on prolonging survival, controlling disease progression, and improving quality of life. This reality underscores the urgent and unmet need for more effective treatment strategies. In the management of advanced breast cancer, chemotherapy-based regimens remain a cornerstone of standard care. However, this approach has reached a significant “therapeutic plateau,” characterized by two major limitations: 1) Limited response rates, wherein current chemotherapeutic agents offer minimal curative potential, particularly in the metastatic setting (3); and 2) Short duration of response, as the benefits of chemotherapy are often transient. Emerging evidence suggests that chemotherapy-induced tissue damage may trigger pro-tumorigenic inflammatory responses, potentially undermining its efficacy and even promoting metastasis and recurrence (4).

Despite advances in existing therapies, the management of advanced breast cancer continues to face formidable challenges. Drug resistance remains a central obstacle, as cancer cells rapidly adapt and develop resistance to treatments (5). For instance, the survival benefit of anti-angiogenic therapies targeting vascular endothelial growth factor receptor 2 (VEGFR2) is limited, in part due to the swift emergence of resistant tumor clones (6). Treatment-related toxicity presents another major constraint. Targeted agents, including anti-angiogenic drugs, are frequently associated with class-specific adverse effects, such as an elevated risk of hypertension and proteinuria (7). These toxicities can impair patients’ quality of life, necessitate dose reductions or interruptions, and ultimately limit the intensity and duration of therapy. In summary, while current therapeutic modalities have achieved some progress, they exhibit pronounced shortcomings in substantially extending OS for patients with advanced breast cancer. The critical barriers of intrinsic and acquired drug resistance, coupled with dose-limiting toxicities, highlight the pressing need to overcome these therapeutic bottlenecks and develop novel, more effective interventions.

Angiogenesis is an established hallmark of tumor development (8). In breast cancer, sustained angiogenesis is critical for tumor growth and metastasis. The abnormal vasculature it generates creates a tumor microenvironment characterized by hypoxia, elevated interstitial fluid pressure, and immune suppression. This aberrant vascular network not only facilitates tumor progression and distant spread but also contributes significantly to therapy resistance (9). Specifically, it can impede the delivery and efficacy of chemotherapeutic agents and targeted drugs while promoting the development of treatment-resistant tumor cell populations through hypoxia-driven selection and immune-editing mechanisms (6). Clinically, the degree of abnormal angiogenesis is strongly correlated with poor prognosis and serves as a key driver of metastatic disease. Inhibitors of angiogenesis primarily function by targeting the VEGF/VEGFR2 signaling axis, a central pathway in tumor vessel growth. Clinically available agents fall into two main categories: mAb (e.g., bevacizumab, which targets VEGFA) and small-molecule TKIs(e.g., sunitinib, sorafenib) (10). While mAb may face challenges related to limited tumor penetration, TKIs can exhibit off-target effects due to their broader kinase inhibition profiles. These agents work by blocking new blood vessel formation, thereby starving the tumor of oxygen and nutrients (11). A significant clinical limitation, however, is the rapid development of resistance to single-agent anti-VEGFR2 therapy, often through the upregulation of alternative pro-angiogenic pathways. The vascular normalization theory offers a refined strategy for combining anti-angiogenic drugs with chemotherapy (12). This paradigm posits that at optimal doses, certain anti-angiogenic agents can “prune” the most immature and dysfunctional tumor vessels, thereby improving the structure and function of the remaining vasculature. In breast cancer, this transient normalization window can enhance blood perfusion, reduce hypoxia, and improve the delivery and efficacy of concurrently administered chemotherapeutic agents. Mechanistic studies suggest that vascular normalization may reversechronic stress-induced vascular dysfunction and potentiate chemotherapy by modulating pathways such as cAMP/PKA/CREB1-mediated glycolysis (13). Furthermore, by alleviating hypoxia and remodeling the tumor microenvironment, vascular normalization can also enhance the efficacy of immunotherapy. Despite its promise, clinical translation faces two major bottlenecks: 1) determining the precise, optimal drug dose and schedule to achieve and maintain the normalization window without inducing excessive vessel pruning, and 2) the lack of reliable, non-invasive biomarkers or imaging techniques to monitor this dynamic window in real-time to guide treatment timing (14).

Current clinical evidence regarding the efficacy of angiogenesis inhibitors in advanced breast cancer is characterized by significant heterogeneity and apparent contradictions. Multiple studies indicate that while anti-angiogenic agents can improve PFS, the benefit in OS is often limited and inconsistent (15). For instance, bevacizumab, the first approved anti-angiogenic drug for breast cancer, has been shown to improve ORR. However, emerging preclinical evidence suggests it may paradoxically activate pro-metastatic pathways such as Wnt/β-catenin signaling, potentially increasing tumor invasiveness and metastasis risk (16). This raises questions about the overall risk-benefit balance, where survival gains may be offset by toxicity risks. Furthermore, comparative efficacy between different drug classes (e.g., mAb vs. TKIs) remains unclear, and conclusions from subgroup analyses—such as potential differential benefits in patients with HER2-positive disease or visceral metastases—are often conflicting (17). The first phase III trial of an anti-angiogenic agent plus a PARP inhibitor in breast cancer showed that the fluzoparib and apatinib group had a median PFS of 11.0 months (HR 0.27 vs chemotherapy). The combination worked better than the single drug (HR 0.60) (18). At the 2025 ASCO annual meeting, a quick oral report said that Hengrui’s rezetuximab (disitamab vedotin) alone or with bevacizumab gave high intracranial response rates (84.4% and 72.7%). This gives a possible new treatment for HER2-positive breast cancer patients with brain metastases. In the context of contemporary, more effective subsequent-line therapies, another critical question arises: does the PFS advantage conferred by angiogenesis inhibitors still translate into a meaningful clinical net benefit for patients? Concurrently, the management and clinical impact of their class-specific toxicities (e.g., hypertension, proteinuria) require systematic evaluation.

This study aims to comprehensively and quantitatively evaluate the efficacy (OS, PFS, ORR) and safety of angiogenesis inhibitors combined with chemotherapy versus chemotherapy alone in advanced breast cancer through a meta-analysis, while exploring potential beneficiary subgroups and influencing factors via pre-specified subgroup analyses. Ultimately, this research is committed to providing high-level evidence-based medical evidence to address current controversies in clinical practice, offering direct and reliable references for clinicians in formulating individualized treatment plans, for researchers in designing subsequent clinical trials, and for health policymakers in optimizing resource allocation.

## Materials and methods

The paper of this systematic review and meta-analysis adhered to the PRISMA guidelines, while its methodological rigor was ensured by following the AMSTAR guidelines.

The methodological quality of this systematic review and meta-analysis was assessed using the AMSTAR checklist. AMSTAR is a validated tool consisting of 16 items that evaluate key aspects of systematic review conduct, including study design, search strategy, data extraction, risk of bias assessment, statistical methods, heterogeneity assessment, and publication bias. This study adhered to the AMSTAR guidelines to ensure methodological rigor and reporting transparency.

### Data sources and search strategy

We systematically searched PubMed and Web of Science using the keywords “Angiogenesis Inhibitor”, “breast cancer”, “Bevacizumab”, “Sunitinib”, “Sorafenib”, and “Anlotinib” for records published up to July 16, 2025, with no language restrictions. This meta-analysis ultimately included 8,480 patients from 29 RCTs. To ensure the inclusion of all currently available RCTs, we also manually screened the reference lists of relevant reviews and meta-analyses. The literature search and selection process were conducted independently by two investigators, with any disagreements resolved through consultation with a third researcher until a consensus was reached.

### Inclusion and exclusion criteria

Studies were eligible if they met the following inclusion criteria: (1)Phase II or III RCTs involving patients with recurrent or metastatic breast cancer confirmed by immunohistochemistry. (2)The experimental group received angiogenesis inhibitors combined with chemotherapy, while the control group received chemotherapy with or without placebo. (3)The primary endpoint was PFS or ORR, and secondary endpoints included CBR, DCR, OS, or safety outcomes.

Only Phase II or III randomized controlled trials were eligible for inclusion. Phase I trials, single-arm studies, observational studies, case reports, and reviews were excluded. This criterion was adopted because Phase II and III RCTs provide higher-quality evidence with adequate sample sizes, randomization, and predefined endpoints, thereby minimizing bias and enabling reliable efficacy and safety comparisons.

The exclusion criteria were as follows: (1) Non-randomized or single-arm clinical trials.(2) Control groups that did not receive chemotherapy combined with placebo.(3) Incomplete or ongoing trials with unpublished results.(4) Unavailable data or inability to extract relevant outcomes.(5) Non-original research, including reviews, systematic reviews, basic research, case reports, meta-analyses, letters, editorials, or expert opinions.

### Data extraction

The data extraction process was conducted in two phases. First, general study characteristics were collected from the included RCTs, including clinical trial registration number, study title, trial phase, year of publication, number of patients in experimental and control groups, tumor stage, treatment regimens in the study and control arms, as well as the dosage and duration of angiogenesis inhibitor therapy. Second, outcome measures were extracted, including PFS, ORR, CBR, DCR, OS, along with corresponding HRs and 95% CIs. Two investigators independently performed the data extraction, with any discrepancies resolved through consultation with a third researcher.

### Literature quality evaluation

All statistical analyses were performed using RevMan (version 5.4, Cochrane Collaboration, Oxford, UK) and Stata (version 17.0, StataCorp, College Station, TX, USA). Hazard ratios (HRs) with 95% confidence intervals (CIs) were pooled for time-to-event outcomes (PFS and OS) using the generic inverse-variance method. Risk ratios (RRs) with 95% CIs were calculated for dichotomous outcomes (ORR, CBR, DCR). Heterogeneity across studies was assessed using the I² statistic and the Cochran Q test. I² values of <25%, 25–50%, and >50% were considered low, moderate, and high heterogeneity, respectively. A random-effects model was applied when I² > 50% or the Q test p-value < 0.10; otherwise, a fixed-effects model was used. Subgroup analyses were performed based on breast cancer subtype (TNBC vs. HR+/HER2–) and drug class (monoclonal antibodies vs. small-molecule TKIs). Publication bias was evaluated using funnel plots and Egger’s regression test when at least 10 studies were available. All p-values were two-sided, and p < 0.05 was considered statistically significant.

## Results

### Literature search results

**Figure 1:**
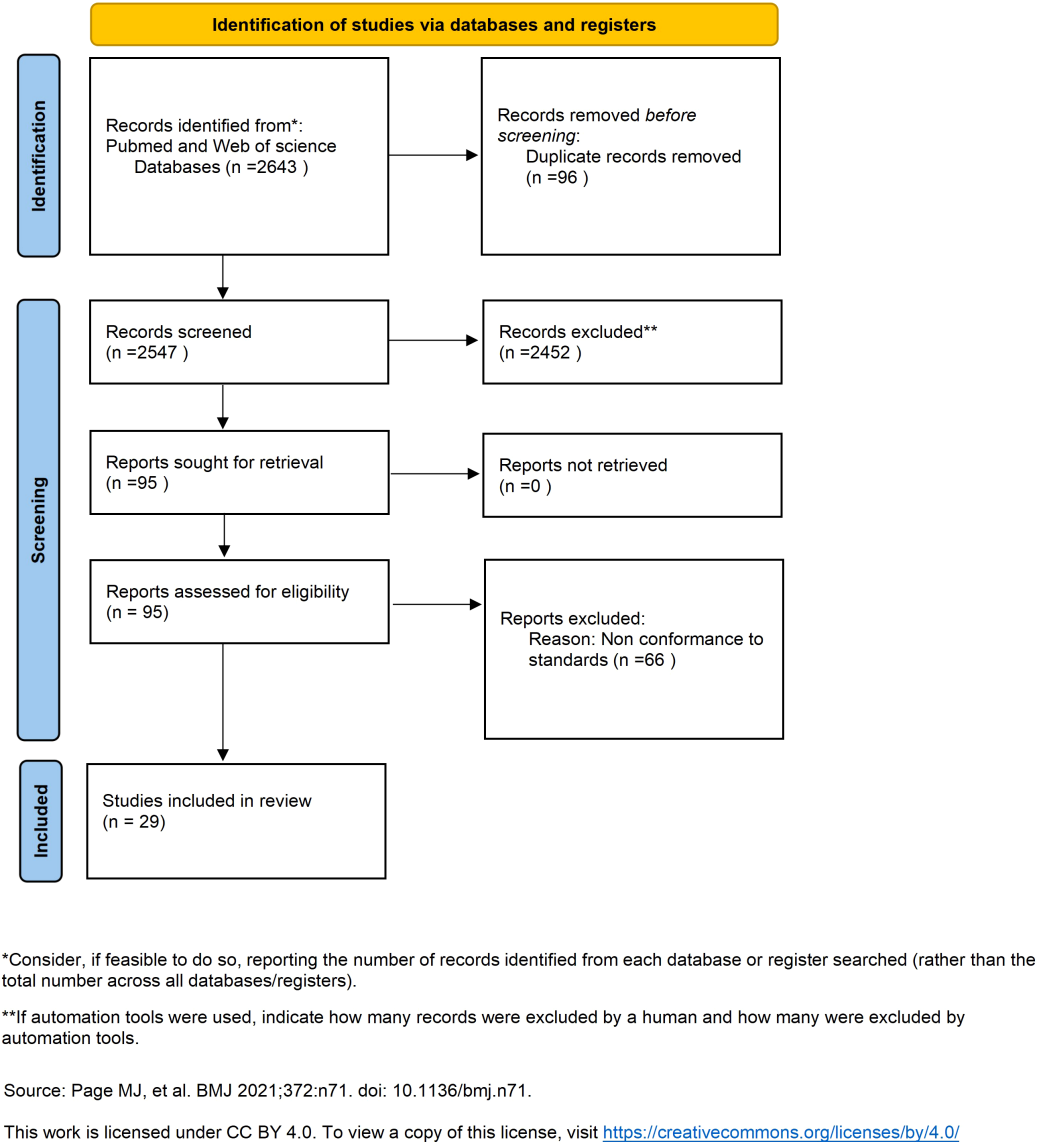
PRISMA 2020 flow diagram for new systematic reviews which included searches of databases and registers only.

The initial database search identified 2,637 non-duplicate records. After title and abstract screening, 2,542 articles were excluded, including reviews, case reports, case series, meta-analyses, retrospective studies, non-RCTs, phase I studies, and trials involving non-breast cancer populations. This resulted in 95 potentially eligible records for full-text review. Following detailed full-text assessment, 66 studies were further excluded due to the following reasons: not evaluating angiogenesis inhibitors, testing monotherapy regimens, or being single-arm clinical trials. After this comprehensive evaluation process, 29 RCTs were ultimately included in the analysis (**Figure 1**).

### Study characteristics and quality assessment

The baseline characteristics of the 29 studies included in this research are detailed in **Table 1**. A total of 11,068 patients with advanced breast cancer were enrolled, all studies being RCTs. Among them, 19 studies employed bevacizumab in the experimental arm, 5 used sorafenib, 2 used sunitinib, 2 utilized anlotinib, and 1 employed ramucirumab.

**Table 1:** Main characteristics of the randomized controlled trials included in the present meta-analysis.

Study	Study design	Primary end point	Secondary end points	Treatment arms	patients, n
NCT00028990 (19)	phase III	PFS	ORR, OS	T T+Bev	354 368
NCT00083031 (20)	phase II	ORR	OS, PFS, safety	CM CM+Bev	21 34
NCT00161291 (21)	phase II	pCR	safety, ORR	L L+Bev	25 50
NCT00356681 (22)	phase II	ORR	PFS, CBR, OS, safety	T T+Bev	94 97
NCT00391092 (23)	phase III	PFS	ORR, OS, safety	T T+Bev	208 216
NCT00433511 (24)	phase III	PFS	OS	AC-T AC+Bev-T+Bev	1000 2008
NCT00435409 (25)	phase III	PFS	ORR, OS, safety	CAP CAP+S	215 217
NCT00499525 (26)	phase II	PFS	ORR, OS, safety	T T+S	119 118
NCT00511459 (27)	phase II	PFS	ORR, OS, safety	T+Tre T+Tre+Bev	56 57
NCT00520975 (28)	phase III	PFS	ORR, OS	T+Cab+H T+Cab+H+Bev	48 48
NCT00545077 (29)	phase III	PFS	ORR, OS, CBR, safety	ET ET-Bev	189 191
NCT00601900 (30)	phase III	PFS	ORR, OS, CBR, safety	L L+Bev	170 173
NCT00785291 (31)	phase III	PFS	OS, safety	T T+Bev	267 275
NCT01186991 (32)	phase III	PFS	ORR, OS	OP OBP	60 63
NCT01234337 (33)	phase III	PFS	ORR, OS, DCR, safety	Cab Cab+S	271 266
NCT01250379 (34)	phase III	PFS	OS, safety	C C+Bev	238 245
NCT01320111 (35)	phase II	PFS	CBR	T T+S	30 30
NCT01427933 (36)	phase II	PFS	ORR, OS, safety	E E+R	70 71
NCT01663727 (37)	phase III	PFS	ORR, OS, safety	T T+Bev	242 239
NCT01898117 (38)	phase II	PFS	OS, safety	CC CC+Bev P P+Bev	13 15 15 15
NCT03254654 (39)	phase II	PFS	ORR, OS, safety	V V+A	32 33
NCT04395989 (40)	phase II	PFS	ORR, OS, DCR	T T+Bev	30 28
NCT05206656 (41)	phase II	PFS	ORR, DCR, safety	E E+A	40 40
PMID: 15681523 (42)	phase III	PFS	ORR, safety	Cap Cap+Bev	230 232
PMID: 20498403 (43)	phase III	PFS	OS	T T+Bev	241 247
PMID: 21990397 (44)	phase III	PFS	ORR, OS, safety	C C+Bev	247 247
PMID: 22331954 (45)	phase III	PFS	ORR, OS, safety	T T+S	297 296
PMID: 22412143 (46)	phase II	PFS	ORR, OS, safety	Cap Cap+S	114 115
PMID: 30822621 (47)	phase II	PFS	OS, safety	T T+S	50 48

T, paclitaxel; Bev, bevacizumab; CM, cyclophosphamide + methotrexate; L, Letrozole; AC-T, doxorubicin + cyclophosphamide - paclitaxel; CAP, capecitabine; SU,sunitinib; S, Sorafenib; Tre, trebananib; Cab, carboplatin; H, trastuzumab; ET, letrozole or fulvestrant; L, letrozole; O, onartuzumab; P,paclitaxe; Cab,capecitabine; S,sorafenib; C, chemotherapy; T,Paclitaxel; S,Sorafenib; E, eribulin; R,ramucirumab; CC, carboplatin + cyclophosphamide; V, Vinorelbine; V+A,Vinorelbine + Apatinib; T,nab-paclitaxel; E, Eribulin; E+A, Eribulin +anlotinib; Cap, capecitabine; T, docetaxel; C, chemotherapy; T, docetaxel; S,sunitinib; Cap, capecitabine; S,+sorafenib.

### Efficacy outcomes

#### CBR

**Figure 2:**
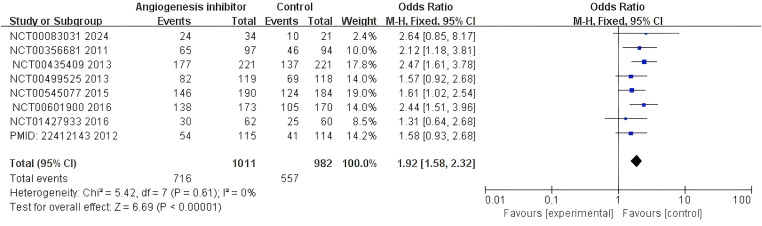
Odds ratio for CBR of angiogenesis inhibitor-based versus angiogenesis inhibitor-free regimens in all included RCTs (the size of the squares is proportional to the weight of each study).

Among the eight studies that reported CBR, a total of 1993 patients were included to evaluate the efficacy of angiogenesis inhibitors compared to the control group in improving the CBR. The random-effects meta-analysis showed that the CBR in the experimental group was significantly higher than that in the control group (OR = 1.92, 95% CI: 1.58 - 2.32, P < 0.001). Low heterogeneity was observed among the studies (I² = 0%, P = 0.61) (**Figure 2**).

#### DCR

**Figure 3:**
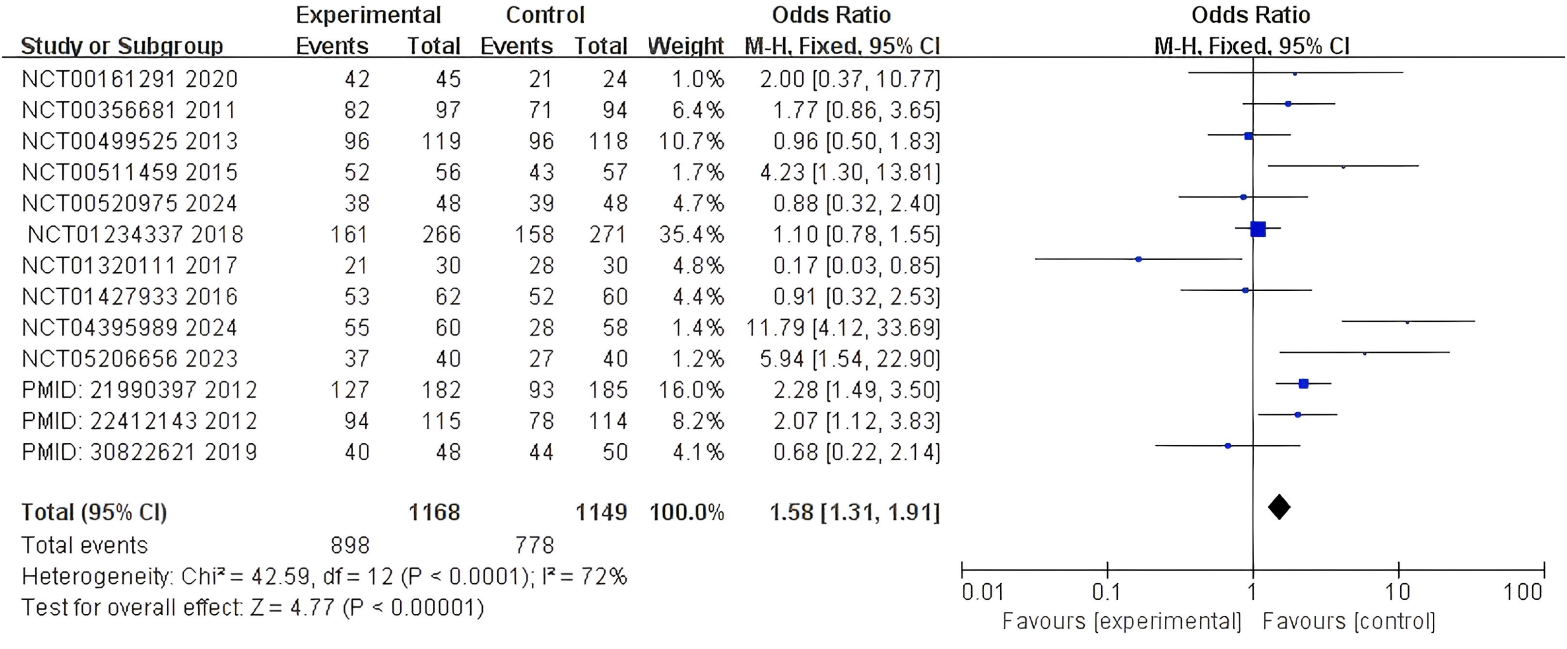
Odds ratio for DCR of angiogenesis inhibitor-based versus angiogenesis inhibitor-free regimens in all included RCTs (the size of the squares is proportional to the weight of each study).

To evaluate the DCR of angiogenesis inhibitors compared to the control group, we conducted a meta-analysis of 13 studies that reported this outcome, involving a total of 2317 patients. The random-effects model analysis showed that the DCR in the experimental group was significantly higher than that in the control group (OR = 1.58, 95% CI: 1.31 - 1.91, P < 0.001) (**Figure 3**). High heterogeneity was observed among the studies (I² = 72%, P < 0.001). In addition, funnel plot analysis indicated no evidence of publication bias (**Supplementary Figure 1**).

#### ORR

**Figure 4:**
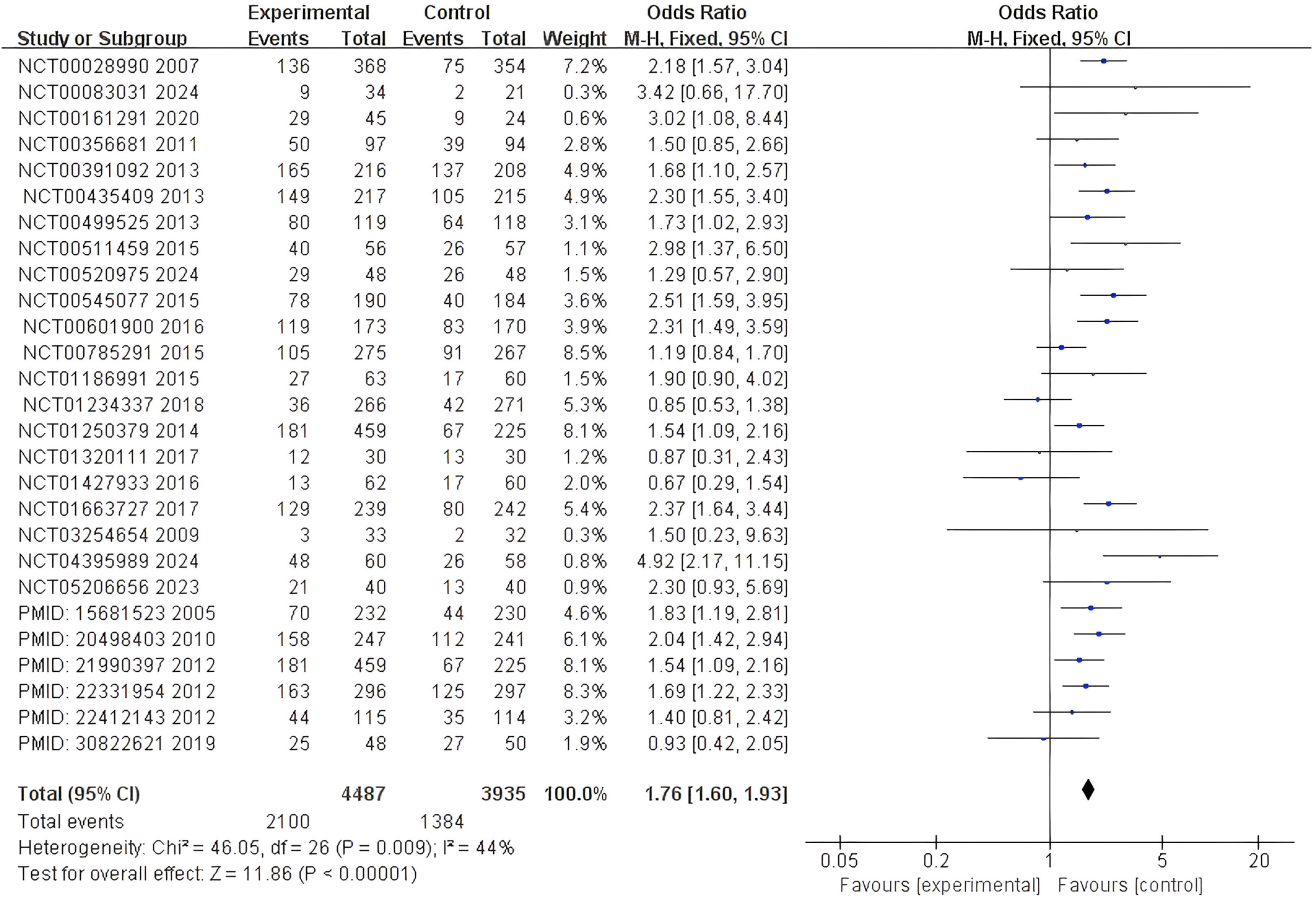
Odds ratio for ORR of angiogenesis inhibitor-based versus angiogenesis inhibitor-free regimens in all included RCTs (the size of the squares is proportional to the weight of each study).

This meta-analysis of 27 included RCTs evaluated the efficacy of angiogenesis inhibitors compared to control in improving the ORR. The analysis included a total of 8422 patients. Random-effects model results showed that the ORR in the experimental group was significantly higher than that in the control group (OR = 1.76, 95% CI: 1.60 - 1.93, P < 0.001) (**Figure 4**). However, moderate heterogeneity was observed among the studies (I² = 44%, P = 0.009). In addition, funnel plot analysis indicated no evidence of publication bias (**Supplementary Figure 2**).

#### PFS

**Figure 5:**
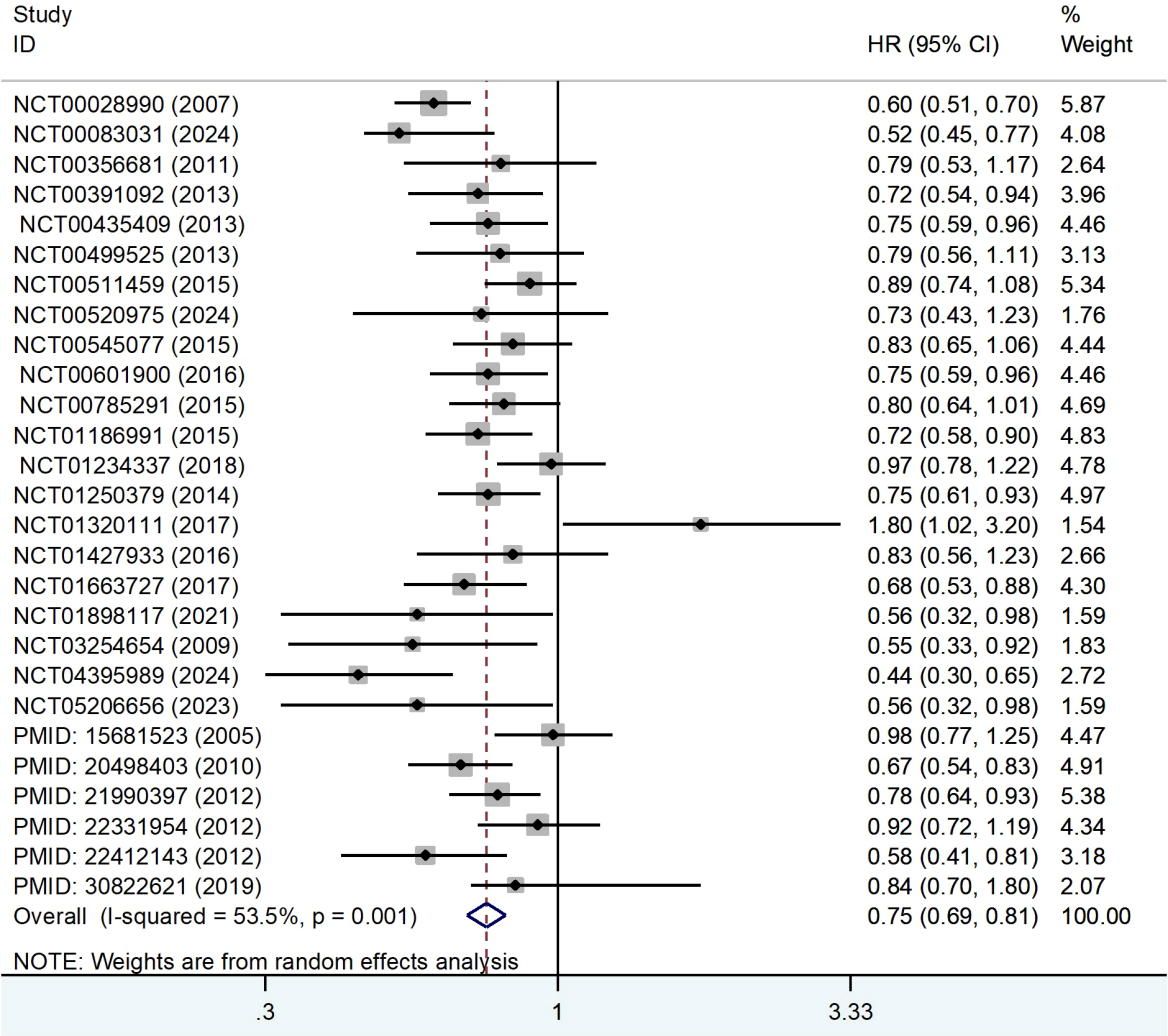
Odds ratio for PFS of angiogenesis inhibitor-based versus angiogenesis inhibitor-free regimens in all included RCTs (the size of the squares is proportional to the weight of each study).

This meta-analysis of 27 RCTs evaluated the efficacy of angiogenesis inhibitors compared to control in prolonging PFS. The random-effects model showed that angiogenesis inhibitors significantly reduced the risk of disease progression or death compared to the control group (HR = 0.75, 95% CI: 0.69-0.81, P < 0.001). Low heterogeneity was observed among the studies (I² = 53.5%, P = 0.001) (**Figure 5**).

#### OS

**Figure 6:**
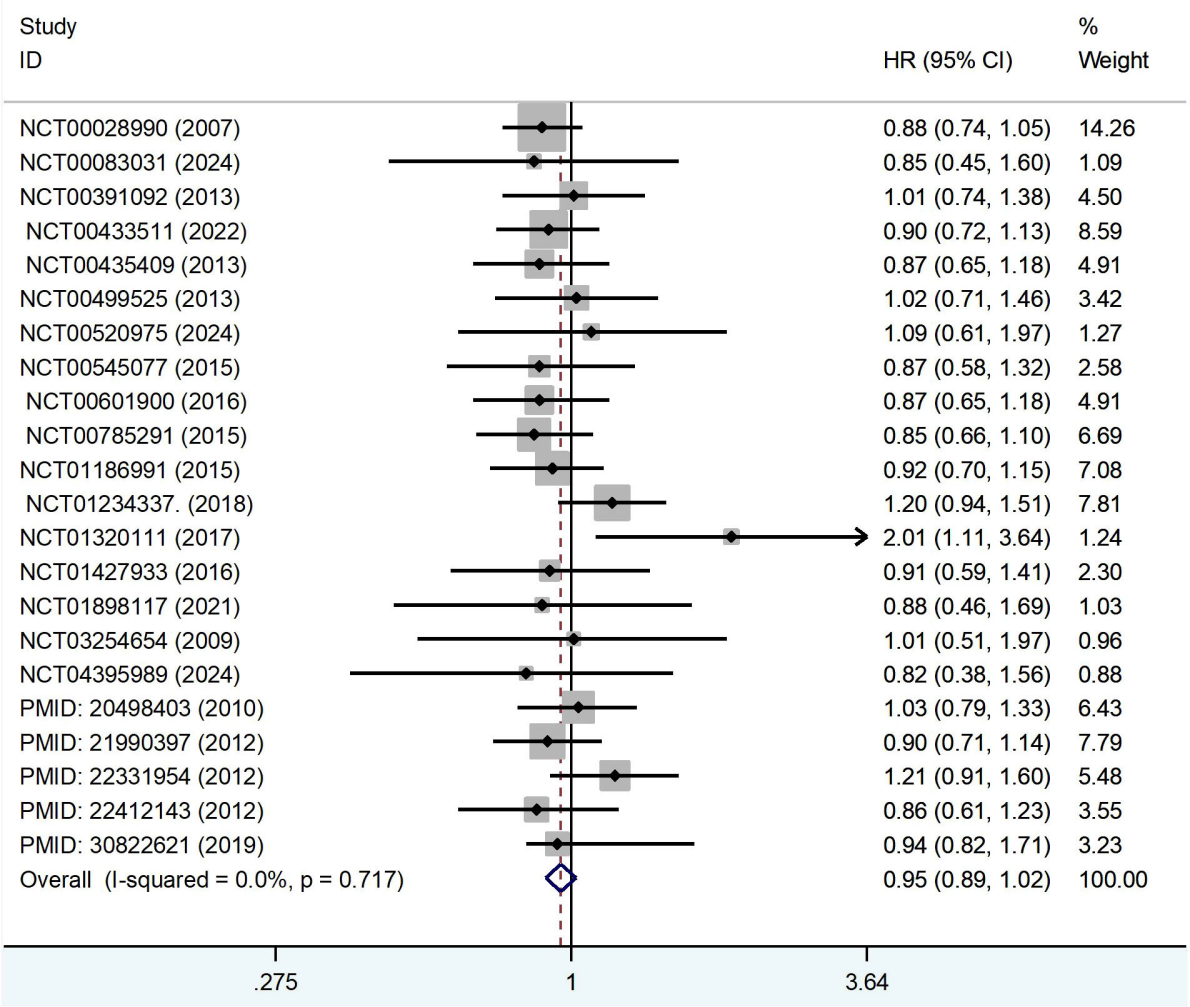
Odds ratio for OS of angiogenesis inhibitor-based versus angiogenesis inhibitor-free regimens in all included RCTs (the size of the squares is proportional to the weight of each study).

We identified 22 studies that evaluated OS in patients with advanced/recurrent breast cancer. The analytical conclusions indicate that, compared to chemotherapy alone, angiogenesis inhibitors did not significantly improve OS (HR 0.95; 95% CI 0.89-1.02; P = 0.171) (**Figure 6**).

### Subtype

**Figure 7:**
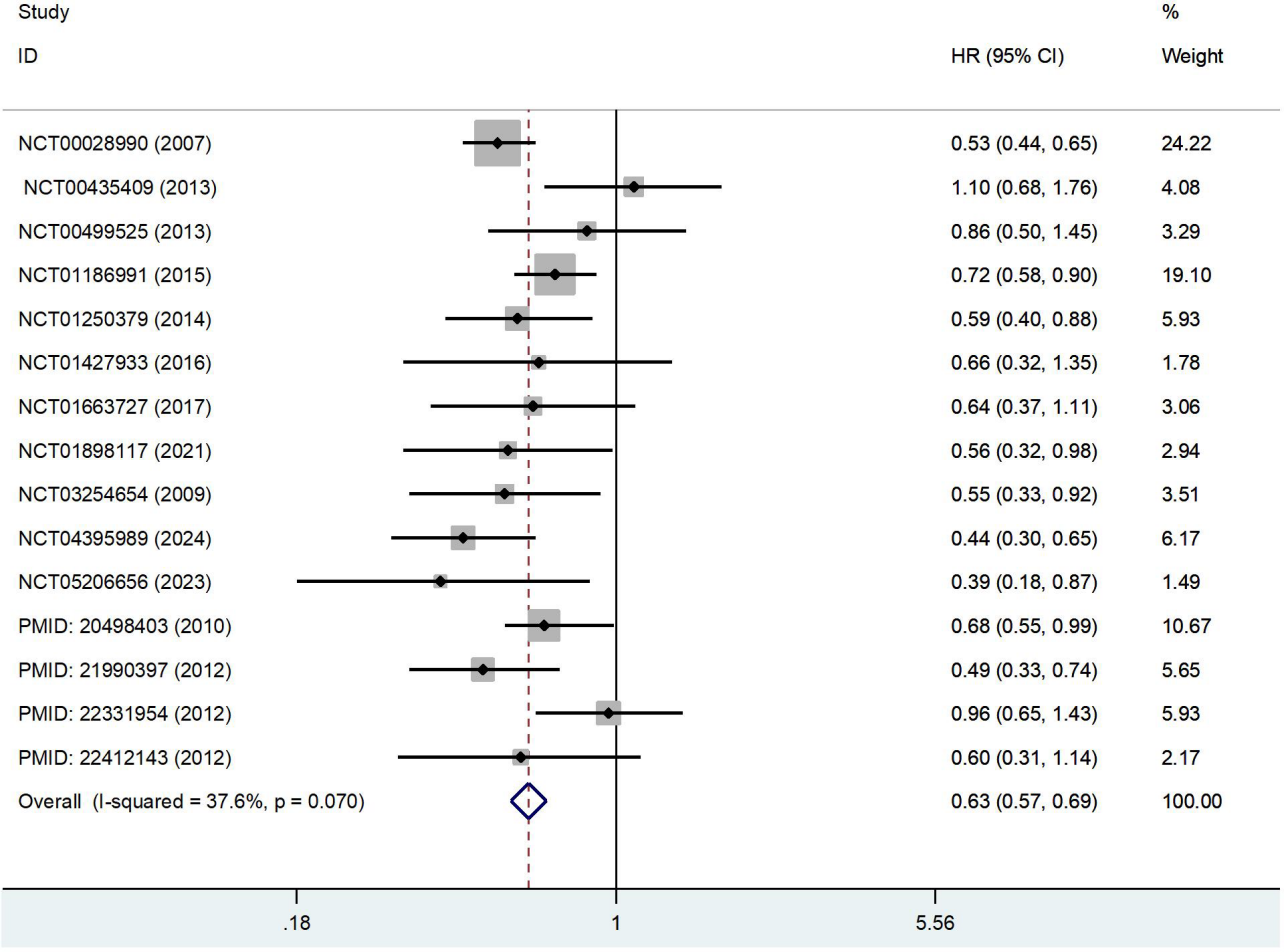
Odds ratio for TNBC PFS of angiogenesis inhibitor-based versus angiogenesis inhibitor-free regimens in all included RCTs (the size of the squares is proportional to the weight of each study).

**Figure 8:**
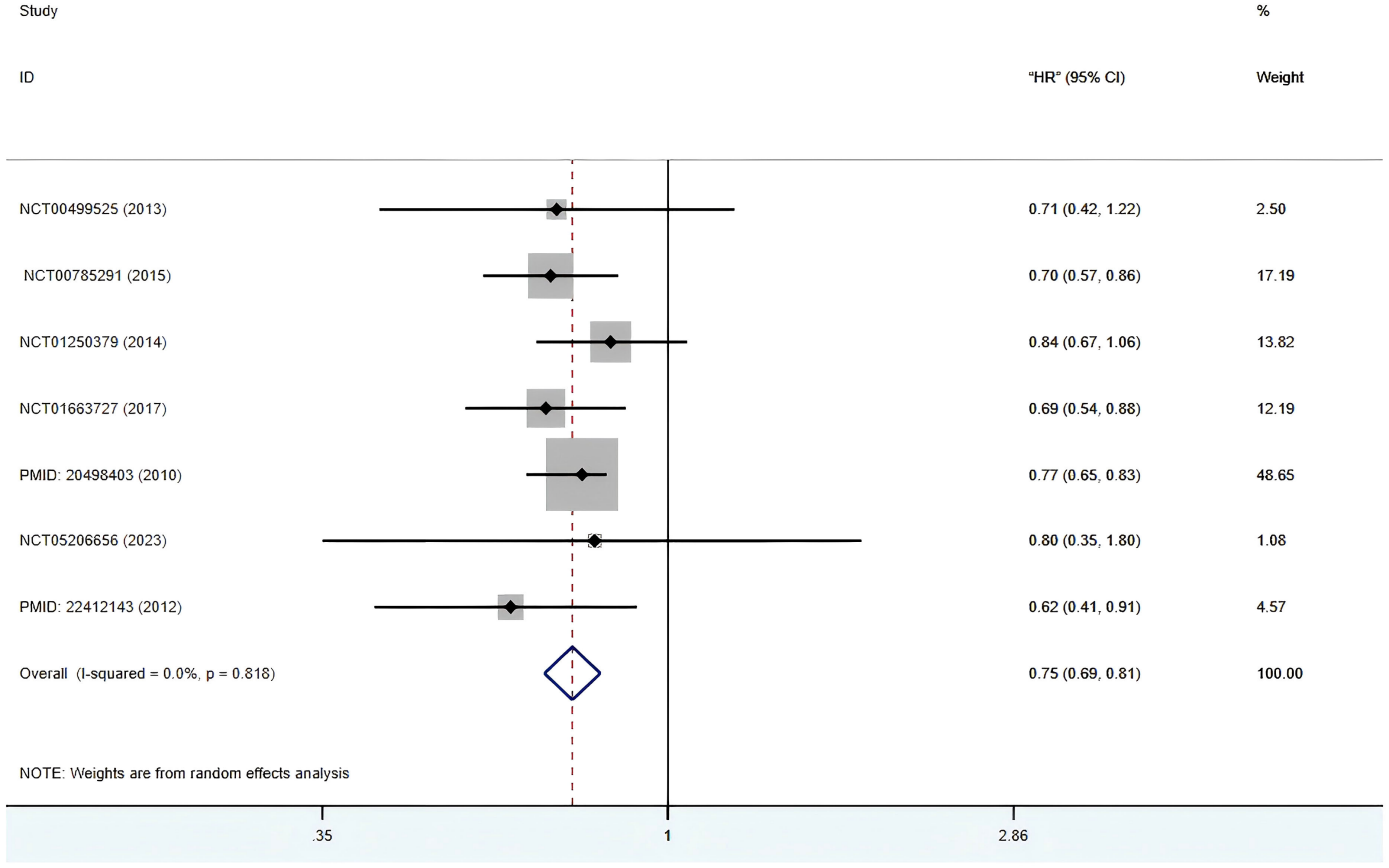
Odds ratio for HR(+) PFS of angiogenesis inhibitor-based versus angiogenesis inhibitor-free regimens in all included RCTs (the size of the squares is proportional to the weight of each study).

This study investigated two breast cancer subtypes. The analysis showed that the addition of angiogenesis inhibitors significantly prolonged PFS in both TNBC and HR(+) breast cancer patients (TNBC: HR = 0.63, (**Figure 7**). HR(+) breast cancer: HR = 0.75) (**Figure 8**).

**Figure 9:**
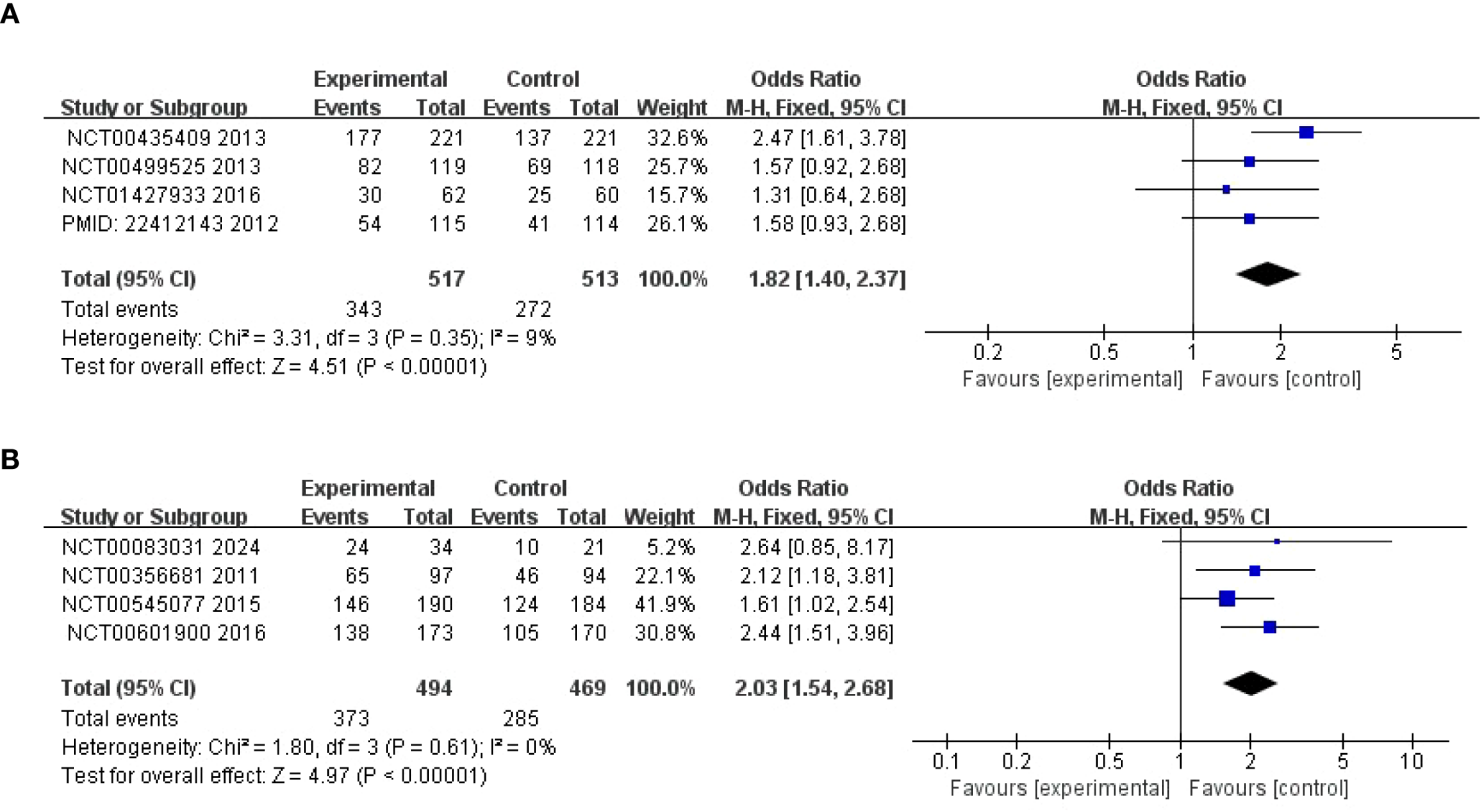
**(A)** Odds ratio for TKI CBR of angiogenesis inhibitor-based versus angiogenesis inhibitor-free regimens in all included RCTs (the size of the squares is proportional to the weight of each study). **(B)** Odds ratio for mAb CBR of angiogenesis inhibitor-based versus angiogenesis inhibitor-free regimens in all included RCTs (the size of the squares is proportional to the weight of each study).

This study analyzed two types of angiogenesis inhibitors. The results showed that for TKIs, the CBR OR = 1.82 (**Figure 9A**). for mAb, the CBR OR = 2.03 (**Figure 9B**).

**Figure 10:**
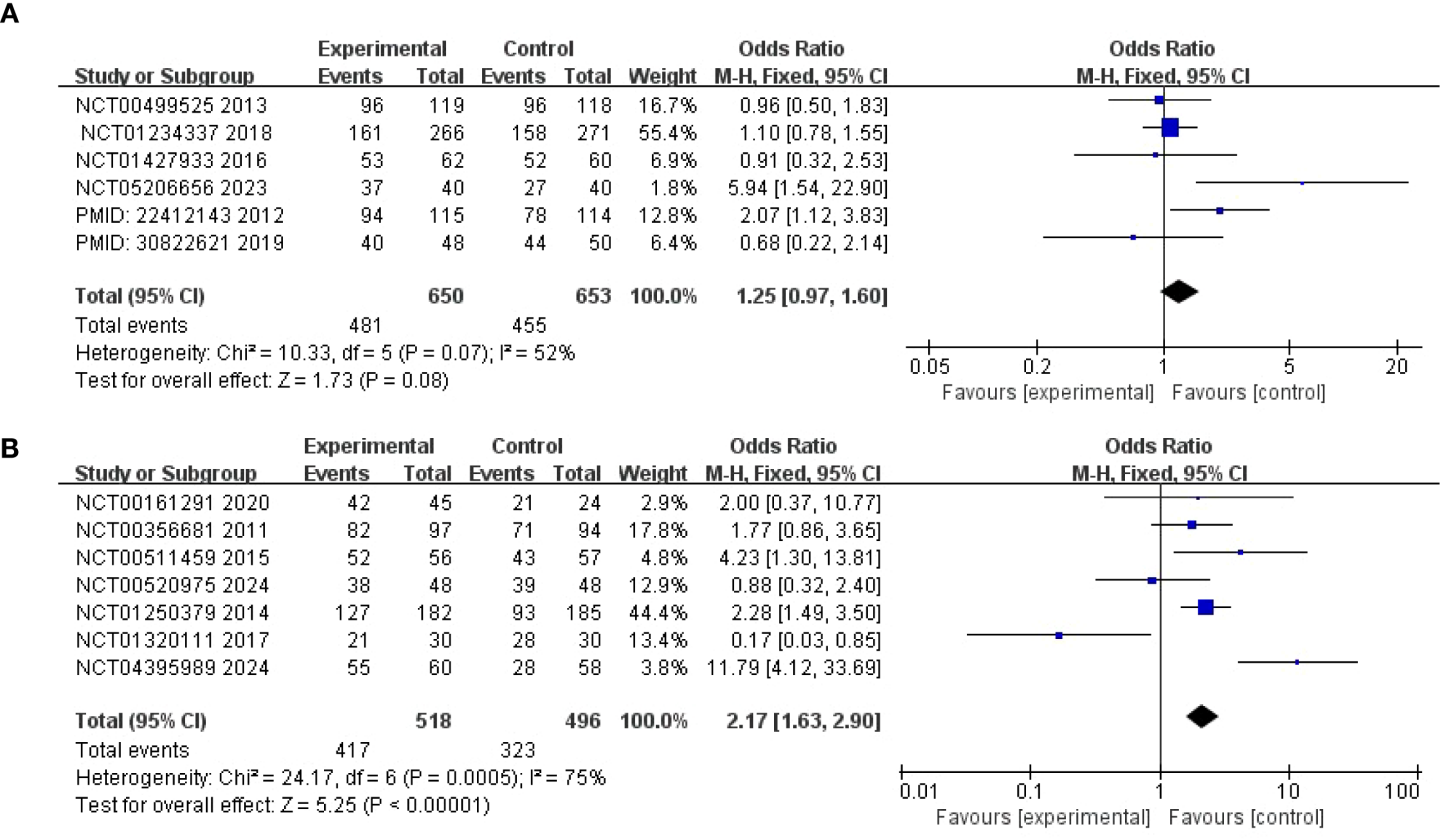
**(A)** Odds ratio for TKI DCR of angiogenesis inhibitor-based versus angiogenesis inhibitor-free regimens in all included RCTs (the size of the squares is proportional to the weight of each study). **(B)** Odds ratio for mAb DCR of angiogenesis inhibitor-based versus angiogenesis inhibitor-free regimens in all included RCTs (the size of the squares is proportional to the weight of each study).

This study analyzed two types of angiogenesis inhibitors. The results showed that for TKIs, the DCR OR = 1.25. (**Figure 10A**) for mAb, the DCR OR = 2.17 (**Figure 10B**).

**Figure 11:**
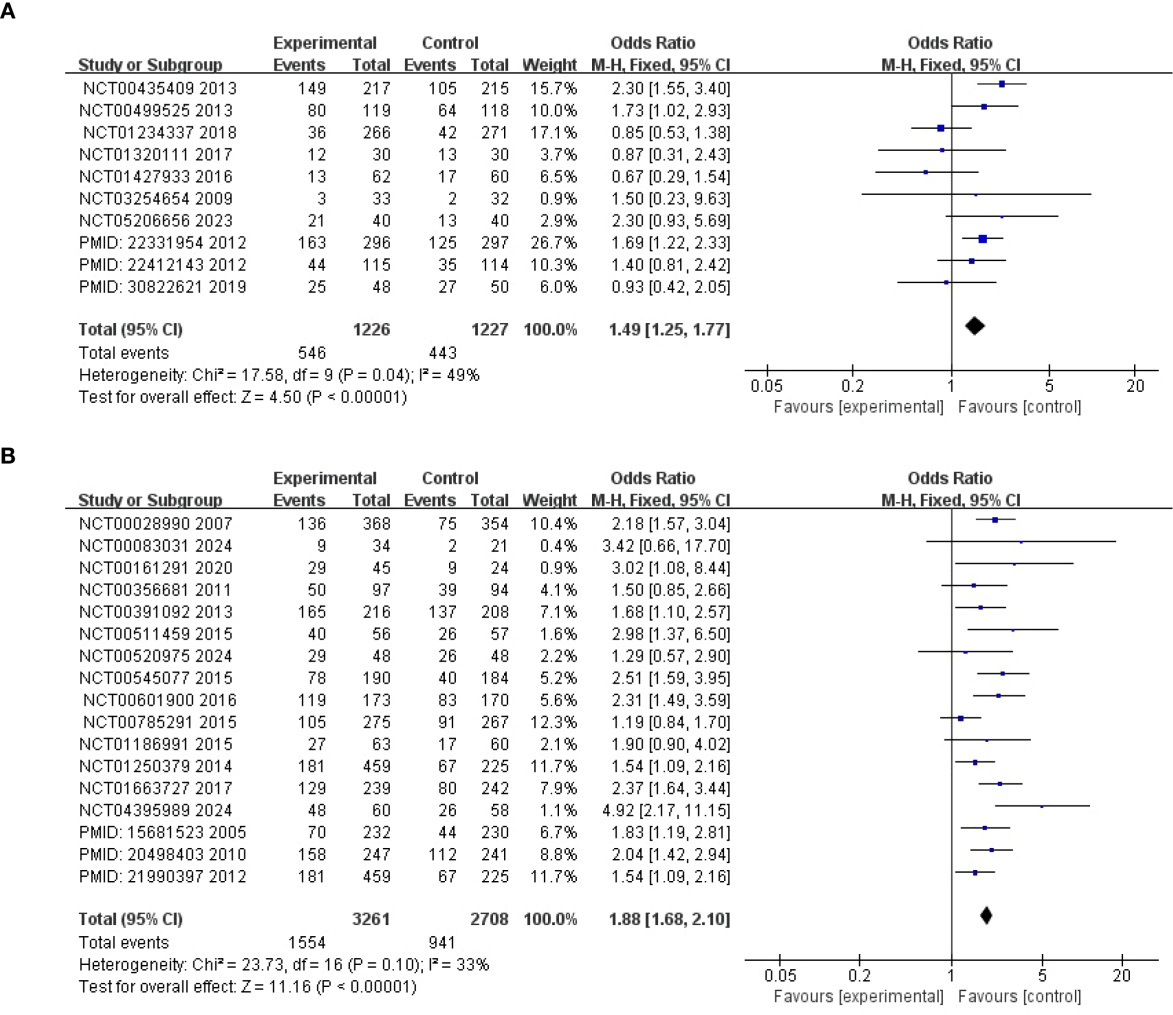
**(A)** Odds ratio for TKI ORR of angiogenesis inhibitor-based versus angiogenesis inhibitor-free regimens in all included RCTs (the size of the squares is proportional to the weight of each study). **(B)** Odds ratio for mAb ORR of angiogenesis inhibitor-based versus angiogenesis inhibitor-free regimens in all included RCTs (the size of the squares is proportional to the weight of each study).

This study analyzed two types of angiogenesis inhibitors. The results showed that for TKIs, the ORR OR = 1.49 (**Figure 11A**) for mAb, the ORR OR = 1.88 (**Figure 11B**).

**Figure 12:**
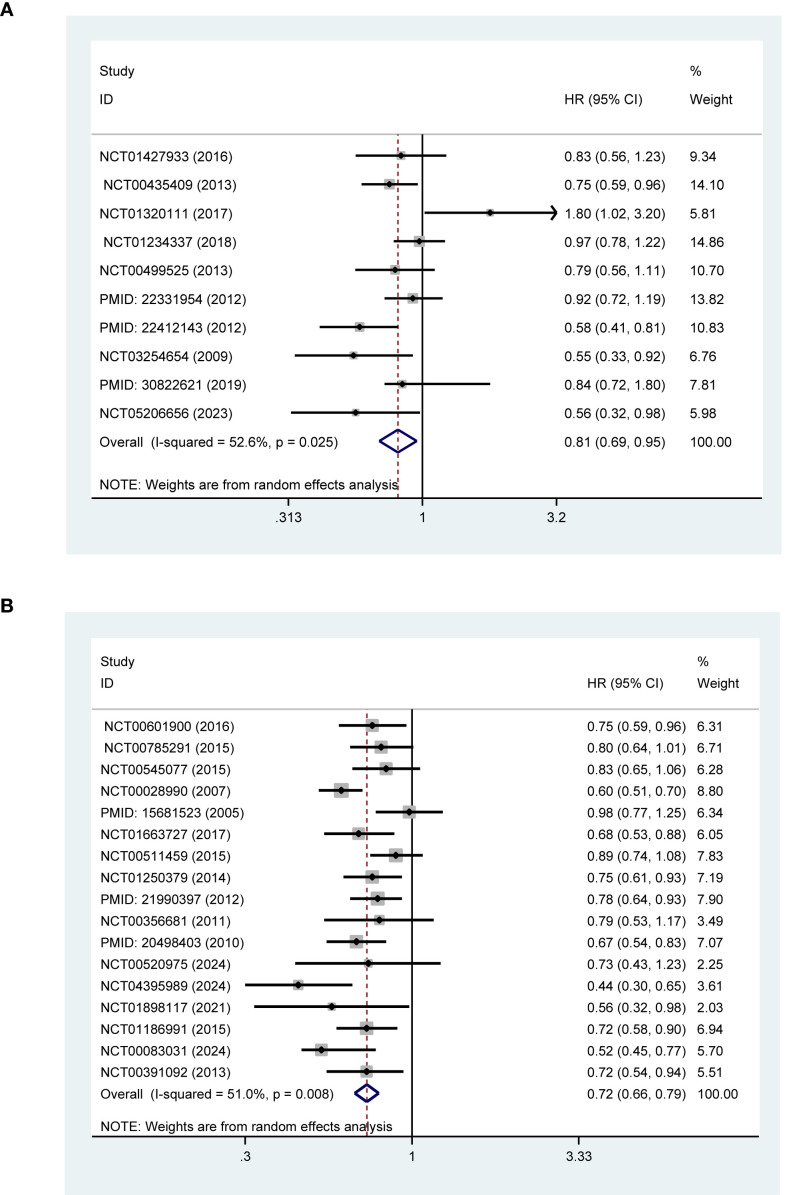
**(A)** Odds ratio for TKI PFS of angiogenesis inhibitor-based versus angiogenesis inhibitor-free regimens in all included RCTs (the size of the squares is proportional to the weight of each study). **(B)** Odds ratio for mAb PFS of angiogenesis inhibitor-based versus angiogenesis inhibitor-free regimens in all included RCTs (the size of the squares is proportional to the weight of each study).

This study analyzed two types of angiogenesis inhibitors. The results showed that for TKIs, the PFS HR = 0.81 (**Figure 12A**) for mAbs, the PFS HR = 0.72 (**Figure 12B**).

**Figure 13:**
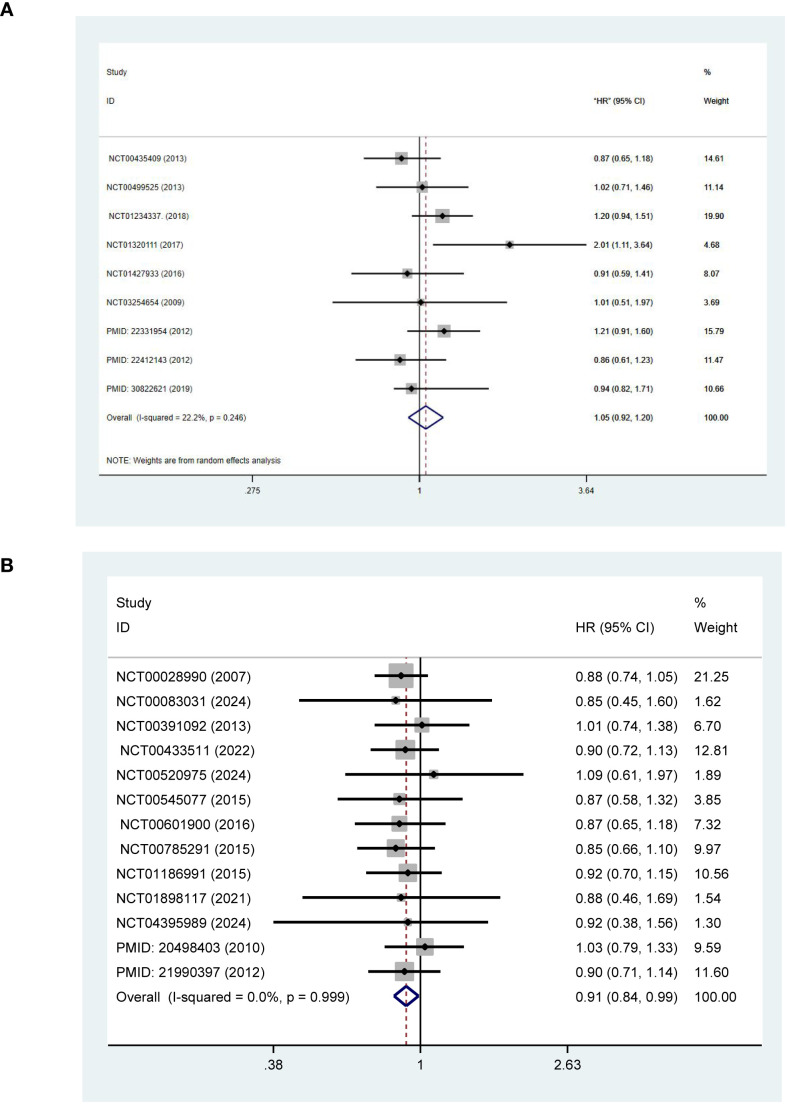
**(A)** Odds ratio for TKI OS of angiogenesis inhibitor-based versus angiogenesis inhibitor-free regimens in all included RCTs (the size of the squares is proportional to the weight of each study). **(B)** Odds ratio for mAb OS of angiogenesis inhibitor-based versus angiogenesis inhibitor-free regimens in all included RCTs (the size of the squares is proportional to the weight of each study).

The results showed that for TKIs, the OS HR = 1.05. (**Figure 13A**). for mAbs, the OS HR = 0.91 (**Figure 13B**).

**Figure 14:**
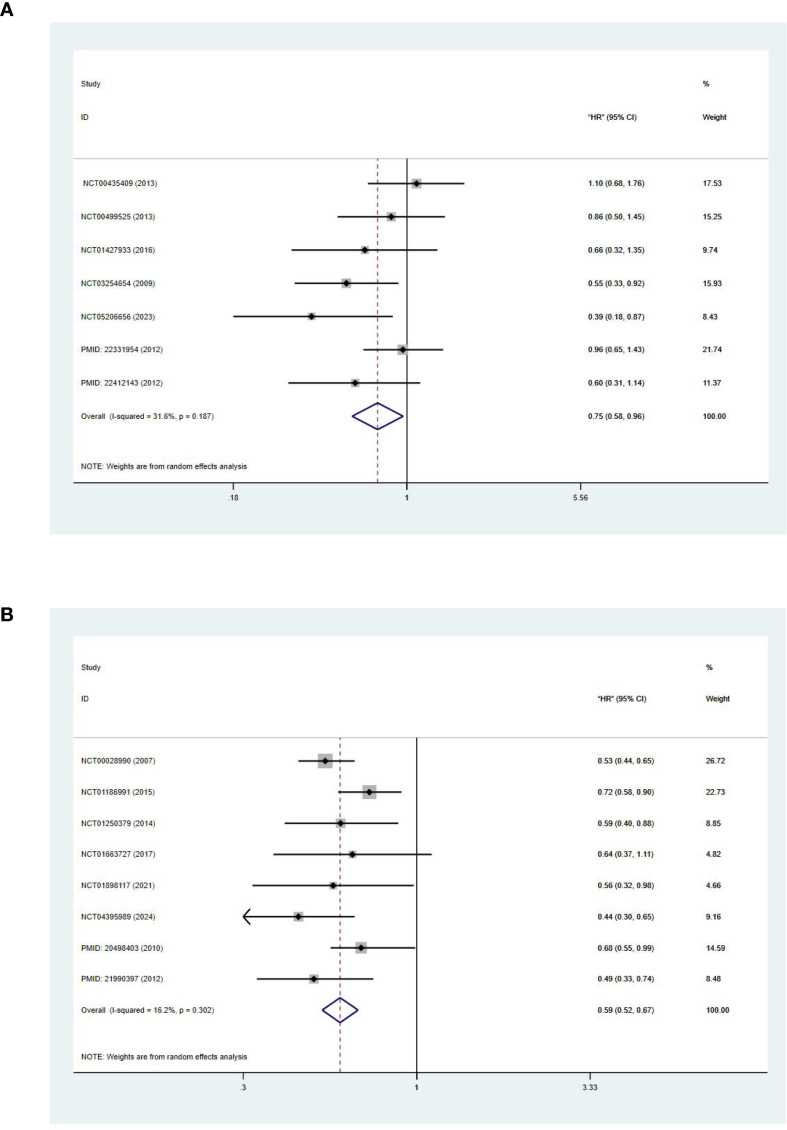
**(A)** Odds ratio for TKI TNBC PFS of angiogenesis inhibitor-based versus angiogenesis inhibitor-free regimens in all included RCTs (the size of the squares is proportional to the weight of each study). **(B)** Odds ratio for mAb TNBC PFS of angiogenesis inhibitor-based versus angiogenesis inhibitor-free regimens in all included RCTs (the size of the squares is proportional to the weight of each study).

The results showed that in TNBC, the PFS HR for TKIs was 0.75 (**Figure 14A**) and the PFS HR for mAbs was 0.59 (**Figure 14B**).

**Figure 15:**
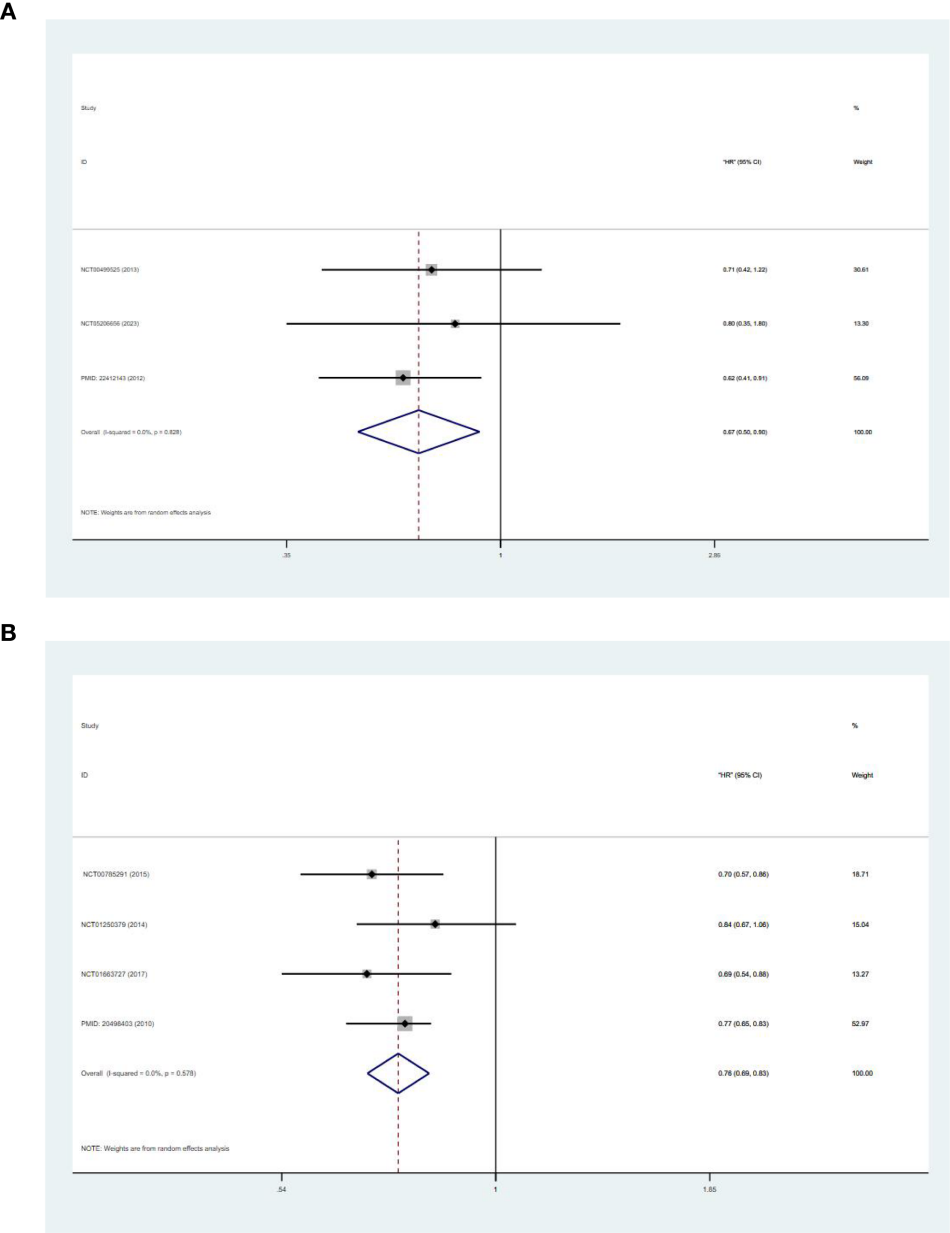
**(A)** Odds ratio for TKI HR(+) PFS of angiogenesis inhibitor-based versus angiogenesis inhibitor-free regimens in all included RCTs (the size of the squares is proportional to the weight of each study). **(B)** Odds ratio for mAb HR(+) PFS of angiogenesis inhibitor-based versus angiogenesis inhibitor-free regimens in all included RCTs (the size of the squares is proportional to the weight of each study).

The results showed that in HR(+) breast cancer, the PFS HR for TKIs was 0.67 (**Figure 15A**) and the PFS HR for mAbs was 0.76 (**Figure 15B**).

### Subgroup analysis

#### Bone metastasis status

**Figure 16:**
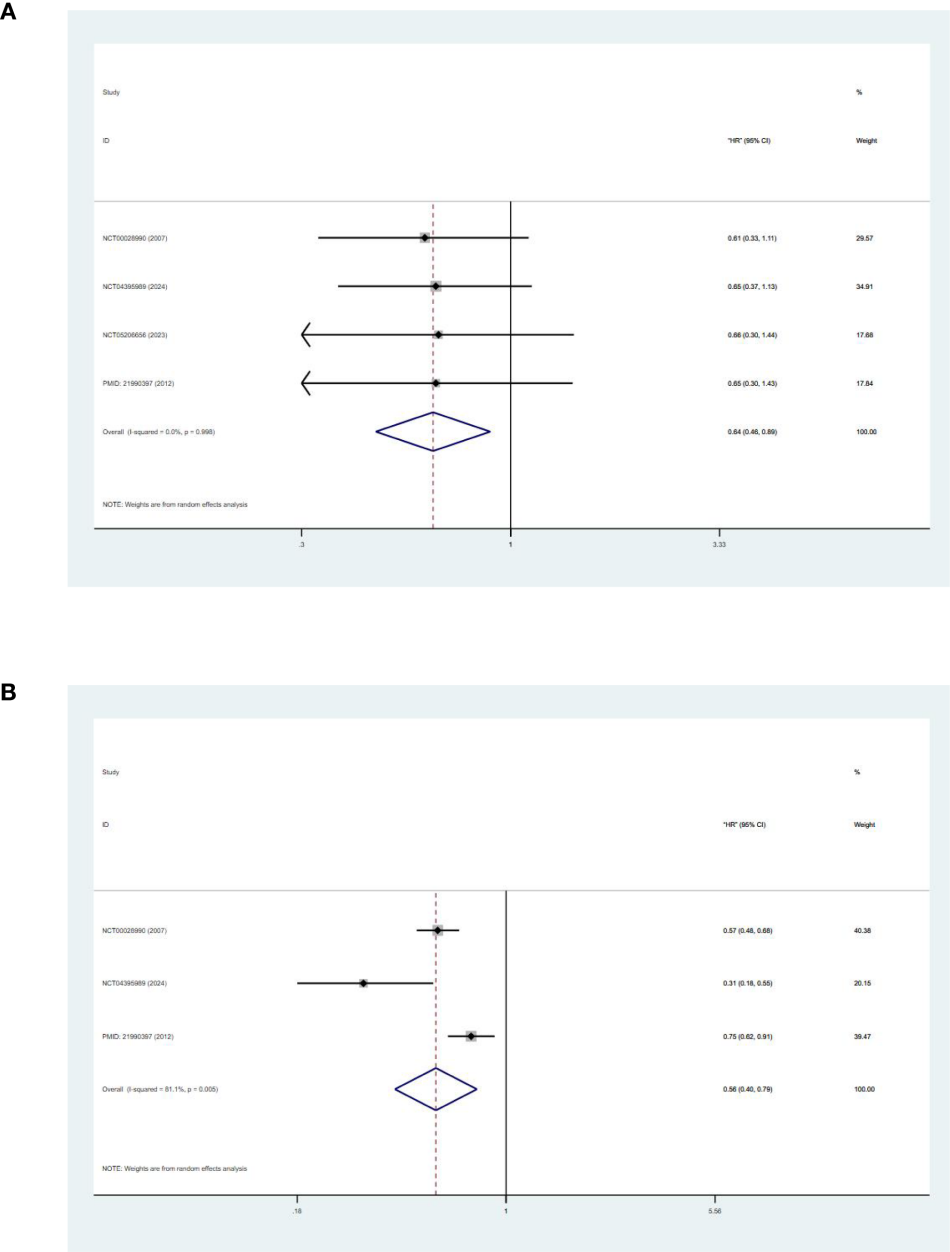
**(A)** Odds ratio for bone metastasis of angiogenesis inhibitor-based versus angiogenesis inhibitor-free regimens in all included RCTs (the size of the squares is proportional to the weight of each study). **(B)** Odds ratio for non-bone metastasis of angiogenesis inhibitor-based versus angiogenesis inhibitor-free regimens in all included RCTs (the size of the squares is proportional to the weight of each study).

Three studies were included in the subgroup of patients without bone metastasis, and four studies were included in the subgroup with bone metastasis. The results demonstrated that angiogenesis inhibitors significantly improved PFS regardless of bone metastasis status (bone metastasis group: HR 0.64. (**Figure 16A**). Bnon-bone metastasis group: HR 0.56. (**Figure 16B**), with potentially greater benefit observed in patients without bone metastasis.

### Visceral metastasis status

**Figure 17:**
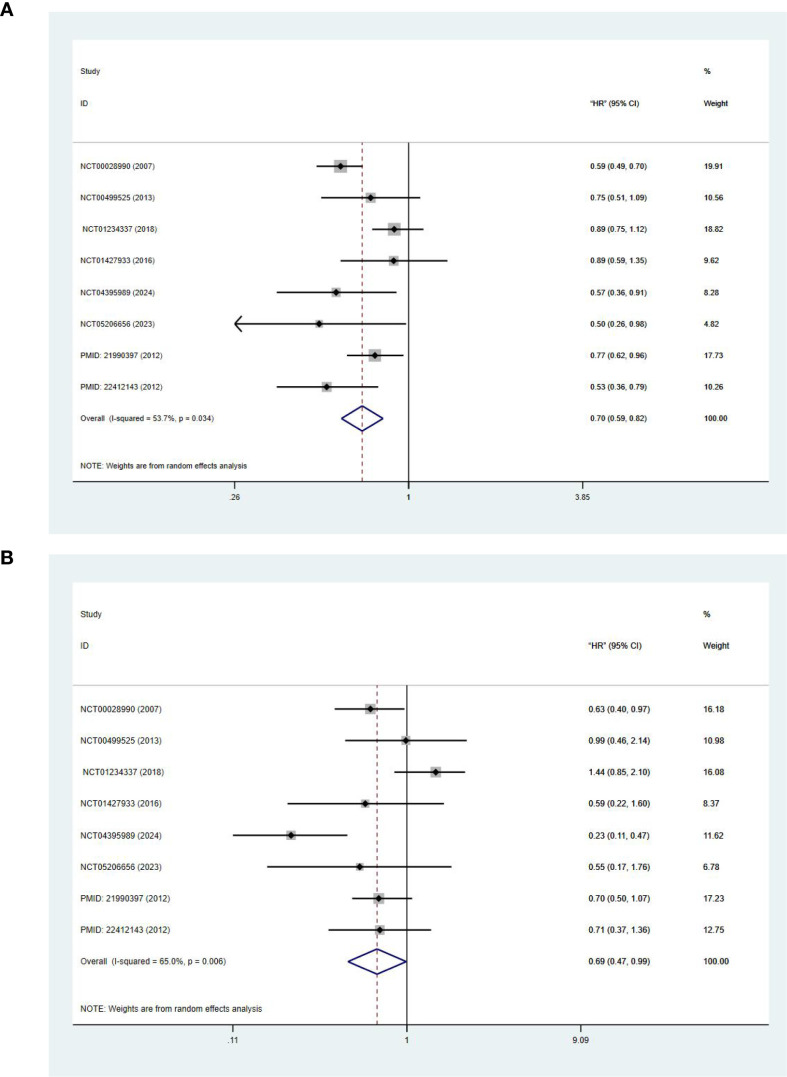
**(A)** Odds ratio for visceral metastasis of angiogenesis inhibitor-based versus angiogenesis inhibitor-free regimens in all included RCTs (the size of the squares is proportional to the weight of each study). **(B)** Odds ratio for non-visceral metastasis of angiogenesis inhibitor-based versus angiogenesis inhibitor-free regimens in all included RCTs (the size of the squares is proportional to the weight of each study).

Eight studies were included in the subgroup of patients with visceral metastasis, and eight studies were included in the subgroup without visceral metastasis. Subgroup analysis revealed that angiogenesis inhibitors significantly improved PFS in both patients without visceral metastasis (HR 0.69) (**Figure 17A**) and those with visceral metastasis (HR 0.70), (**Figure 17B**) with highly consistent risk reduction magnitudes (approximately 30%). This indicates that visceral metastasis status does not affect the intrinsic antitumor activity of this treatment regimen.

### No. of metastasis sites

**Figure 18:**
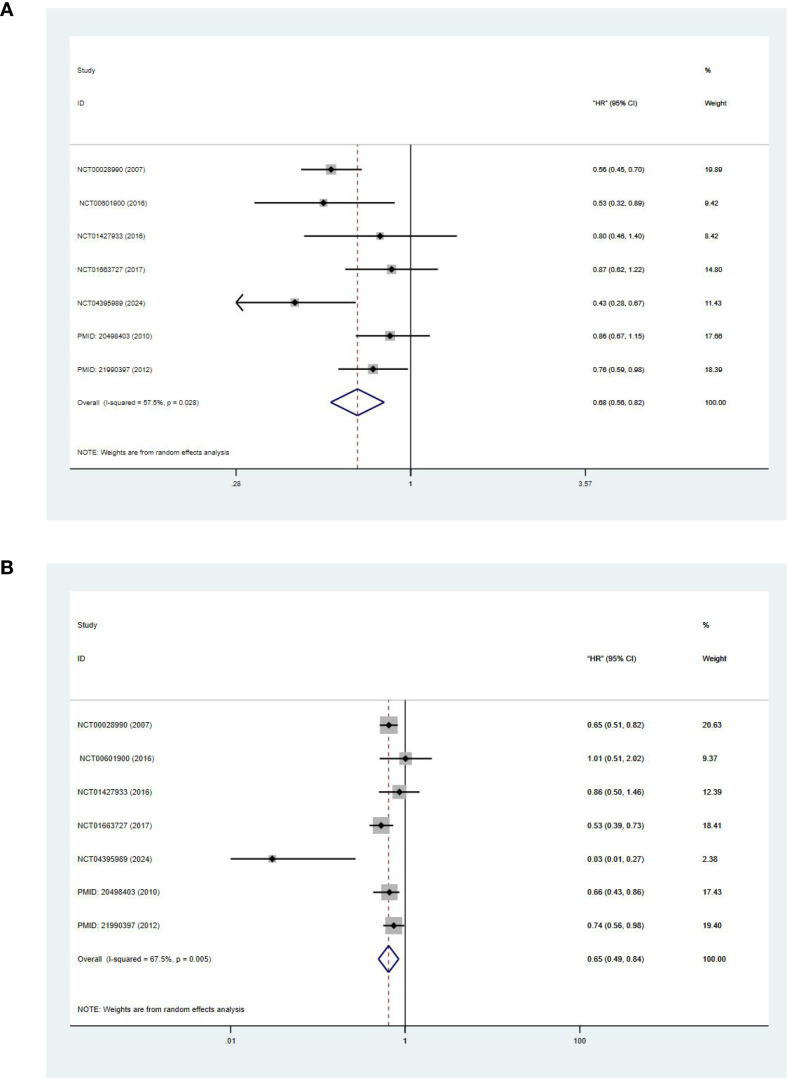
**(A)** Odds ratio for No. of sites 0–3 of angiogenesis inhibitor-based versus angiogenesis inhibitor-free regimens in all included RCTs (the size of the squares is proportional to the weight of each study). **(B)** Odds ratio for No. of sites >3 of angiogenesis inhibitor-based versus angiogenesis inhibitor-free regimens in all included RCTs (the size of the squares is proportional to the weight of each study).

Seven studies were included in the subgroup of patients with ≤3 metastatic sites, and seven studies were included in the subgroup with >3 metastatic sites. Subgroup analysis demonstrated that angiogenesis inhibitors significantly improved PFS regardless of the number of metastatic sites (≤3 vs >3: HR 0.68 vs 0.65), (**Figure 18**). with highly consistent risk reduction (approximately 35%). These findings indicate that this treatment regimen remains equally effective in patients with high tumor burden, demonstrating universal efficacy independent of metastatic site number.

### Safety outcome

We analyzed the safety of angiogenesis inhibitors combined with chemotherapy versus chemotherapy alone in patients with advanced or recurrent breast cancer. Overall, the incidence of adverse events (AEs) in the angiogenesis inhibitor combination group was 1.47 times higher than in the chemotherapy-alone group (OR = 1.47). (**Supplementary Figure 3**). The incidence rates of most other selected AEs showed no significant differences between the two groups. However, we found that the following AEs occurred at significantly higher rates in the angiogenesis inhibitor combination group: anorexia (OR = 1.82), (**Supplementary Figure 4**). diarrhea (OR = 1.97), (**Supplementary Figure 5**) hand-foot syndrome (OR = 2.14), (**Supplementary Figure 6**) headache (OR = 2.11), (**Supplementary Figure 7**) hypertension (OR = 4.59), (**Supplementary Figure 8**). neutropenia (OR = 1.64), (**Supplementary Figure 9**). proteinuria (OR = 2.38), (**Supplementary Figure 10**) and thrombocytopenia (OR = 4.54) (**Supplementary Figure 11**).

## Discussion

This study synthesizes the latest evidence from 29 RCTs to systematically evaluate the efficacy and safety of combining angiogenesis inhibitors with chemotherapy in advanced breast cancer. Our analysis not only confirms the definitive value of this combination strategy in enhancing tumor control metrics but also, through in-depth subgroup analyses, reveals underlying patterns of efficacy variation. Furthermore, it provides a critical foundation for informing personalized clinical treatment decisions.

The most pivotal finding of this meta-analysis is that angiogenesis inhibitors, when combined with chemotherapy, can improve disease control in patients with advanced breast cancer across multiple dimensions—significantly enhancing the CBR, DCR, ORR, and prolonging PFS. This outcome robustly supports the clinical translational value of the “vascular normalization” theory: appropriate vascular-targeted therapy can modify the tumor microenvironment and enhance the delivery efficiency of chemotherapeutic agents (48), thereby generating synergistic anti-tumor effects (12). Notably, in the most aggressive TNBC subtype, we observed the most pronounced PFS benefit. This is closely associated with the typically hyperactive angiogenic phenotype of this subtype (49, 50). Although OS did not demonstrate a significant extension, in the current era of multi-line sequential therapy, the significant prolongation of PFS itself holds substantial clinical significance—it provides patients with the opportunity to receive subsequent novel treatments and maintains a better quality of life during this period.

Our subgroup analyses revealed efficacy differences among patients with varying metastatic characteristics, a finding of profound biological implication and clinical guidance value. Subgroup analyses were conducted for two breast cancer subtypes: TNBC and HR(+)/HER2(-) breast cancer. These subtypes were selected because they represent the majority of HER2(-) breast cancer cases, have distinct biological features, and had sufficient patient numbers across the included trials for meaningful subgroup comparisons. HER2(+) breast cancer was excluded due to the confounding effect of concurrent anti-HER2 therapy. First, the phenomenon that patients without bone metastases derived more significant benefit likely reflects two distinct tumor microenvironment types: the unique osteoblast-osteoclast interactions within bone metastasis niches may create a distinctive immunosuppressive and pro-angiogenic ecological niche through the secretion of cytokines such as IL-6 and TGF-β, thereby attenuating the effect of exogenous vascular-targeted agents (51) (52).Second, the finding that visceral metastasis status and the number of metastatic sites had minimal impact on anti-tumor activity is particularly crucial. It suggests that the mechanism of action of angiogenesis inhibitors is broad-spectrum, with its efficacy not constrained by tumor burden size. This provides clinicians with significant confidence—even patients with extensive metastases may still derive substantial benefit from combination therapy, provided their biological characteristics align (e.g., absence of bone metastases). Finally, we identified significant efficacy heterogeneity across different drug classes and breast cancer subtypes. In the overall population, mAb (e.g., bevacizumab) demonstrated more robust benefits in both PFS and OS compared to small-molecule TKIs (PFS HR 0.72 vs. 0.81; OS HR 0.91 vs. 1.05). This difference was particularly pronounced in advanced TNBC, where the efficacy advantage of mAb was evident (HR 0.59 vs. 0.75). Conversely, in HR(+) breast cancer, TKIs showed a superior point estimate of efficacy (HR 0.67 vs. 0.76), suggesting they may more effectively inhibit bypass signaling pathways associated with endocrine resistance in this subtype. The results above strongly indicate that angiogenesis inhibitors are not a homogeneous therapeutic option. Clinical decision-making should be differentiated based on molecular subtype and drug class: mAb should be prioritized in TNBC, while TKIs may represent a potential optimized choice for specific subgroups within HR(+) breast cancer.

Potential pro-metastatic effects of angiogenesis inhibitors have been described in preclinical and clinical studies. Mechanistically, anti-angiogenic therapy-induced hypoxia can stabilize HIF-1α and HIF-2α (53), leading to upregulation of EMT-related genes (e.g., Snail, Twist, MMPs) and enhanced tumor cell invasion and intravasation. Additionally, chronic VEGF blockade may select more aggressive tumor clones that utilize alternative pro angiogenic pathways associated with aggressive phenotypes (54). Clinically, while some trials have reported improved PFS with angiogenesis inhibitors, the absence of OS benefit and potential early increases in distant metastases in certain subgroups warrant cautious interpretation. These findings highlight the need for careful patient selection, optimized dosing schedules, and combination strategies to mitigate potential pro-metastatic risks.

This study systematically delineated the specific toxicity profile of angiogenesis inhibitors. AEs associated with VEGF pathway inhibition, such as hypertension and proteinuria, along with chemotherapy-overlapping toxicities like myelosuppression and gastrointestinal reactions, necessitate the establishment of a comprehensive clinical framework for proactive monitoring and graded management. Based on our analysis, we propose the following structured recommendations for clinical practice: 1) Pre-Treatment Baseline Assessment: A thorough evaluation prior to initiation of therapy is mandatory. This assessment must include a detailed evaluation of cardiovascular risk factors, baseline renal function, and screening for pre-existing proteinuria. 2) Intensified Monitoring During Initial Treatment: Vigilant monitoring is crucial, particularly during the first two treatment cycles. Blood pressure should be monitored closely, and prophylactic or early intervention with antihypertensive agents should be implemented based on established guidelines to prevent severe hypertension. 3) Dynamic Monitoring Protocol for Proteinuria: A protocol for the dynamic monitoring of urine protein levels should be established. Timely intervention, which may include dose modification or temporary suspension of the angiogenesis inhibitor, is required for persistent or Grade 2 (or higher) proteinuria according to Common Terminology Criteria for AEs (CTCAE) grading (55). Furthermore, special attention must be paid to toxicities that profoundly impact quality of life and treatment adherence, such as hand-foot syndrome (palmar-plantar erythrodysesthesia) and diarrhea. Proactive patient education regarding these potential AEs and the pre-emptive formulation of supportive care plans for symptom management are essential components of patient care. In conclusion, the meticulous optimization of these toxicity management strategies is not merely supportive but a critical determinant of therapeutic success. It ensures that patients can tolerate and sustainably receive the full intended course of effective combination therapy, thereby creating the necessary conditions to ultimately translate treatment efficacy into maximized clinical benefit.

This study has several limitations. First, although we attempted to conduct subgroup analyses on key factors such as chemotherapy regimens and lines of therapy, no statistically significant subgroup analysis results were ultimately obtained due to excessive heterogeneity among studies and the presence of other confounding factors. Second, the majority of included studies lacked detailed biomarker information, which limited our ability to identify the specific patient subgroups most likely to derive maximum benefit from the treatment. Third, a limited number of studies (8/29) reported CBR data. This is largely because CBR is not a mandatory endpoint in most angiogenesis inhibitor trials, and definitions of CBR varied across studies, particularly regarding the minimum duration of stable disease. Additionally, many trials prioritized ORR and PFS as primary or secondary endpoints without systematically collecting or reporting the data necessary for CBR calculation. Based on these findings, future research should focus on the following directions: 1) Develop Predictive Biomarkers: Efforts should be concentrated on biomarker research based on circulating tumor DNA (ctDNA), dynamic angiogenic factor profiles, or radiomic features, aiming to construct precise patient selection models for treatment individualization. 2) Design randomized trials comparing different categories of angiogenesis inhibitors (such as mAb and TKIs) to clarify their optimal respective application scenarios. 3) Explore Novel Combination Therapeutic Strategies: It is worthwhile to investigate triple-combination regimens involving angiogenesis inhibitors, immune checkpoint inhibitors, and chemotherapy. This strategy aims to achieve a breakthrough in synergistic antitumor effects by simultaneously modulating both the vascular and immune tumor microenvironments.

## Conclusion

This meta-analysis based on 29 RCTs demonstrated that the combination of angiogenesis inhibitors and chemotherapy could improve the CBR, DCR, ORR, and PFS in patients with advanced breast cancer. Particularly in advanced TNBC, the PFS benefit was more pronounced, although OS was not prolonged. Further analysis revealed that compared to chemotherapy alone, patients without bone metastases derived significantly greater benefit from angiogenesis inhibitor combination therapy. Visceral metastasis status and the number of metastatic sites had minimal impact on antitumor activity. Monoclonal antibodies should be prioritized for use in TNBC, whereas TKIs may represent a potential optimized choice for specific subgroups with HR(+) breast cancer. Additionally, closer monitoring of adverse reactions in patients receiving angiogenesis inhibitors is warranted, including anorexia, diarrhea, hand-foot syndrome, headache, hypertension, neutropenia, proteinuria, and thrombocytopenia.

## Data Availability

Publicly available datasets were analyzed in this study. This data can be found here: Pubmed and Web of science.
