# Correction: The efficacy of angiogenesis inhibitors combined with chemotherapy in advanced breast cancer: a systematic review and meta-analysis

**DOI:** 10.3389/fonc.2026.1901329

**Published:** 2026-06-15

**Authors:** Jiangzhuo Wu, Hanbing Li, Ling Wei, Xiao Yan, Jiang Fang, Lin Peng, Xiaobo Zhao

**Affiliations:** 1 Department of Thyroid and Breast Surgery, Affiliated Hospital of North Sichuan Medical College, Nanchong, Sichuan, China; 2 National Clinical Key Specialty (General Surgery)/National Clinical Research Center for Digestive Diseases/Sichuan Clinical Research Center For Digestive Diseases, Nanchong, Sichuan, China

**Keywords:** angiogenesis inhibitors, breast cancer, chemotherapy, meta-analysis, survival

Affiliation “Department of Thyroid and Breast Surgery, Affiliated Hospital of NorthSichuan Medical College, Nanchong, Sichuan, China” was omitted for author “Jiangzhuo Wu, Hanbing Li, Ling Wei, Xiao Yan, Jiang Fang, Lin Peng, and Xiaobo Zhao”. This affiliation has now been added for author “Jiangzhuo Wu, Hanbing Li, Ling Wei, Xiao Yan, Jiang Fang, Lin Peng, and Xiaobo Zhao”.

The original version of this article has been updated.

