# Supplementary material for: The efficacy of angiogenesis inhibitors combined with chemotherapy in advanced breast cancer: a systematic review and meta-analysis
Source: Front Oncol. 2026 Jun 5;16:1820120. doi: 10.3389/fonc.2026.1820120 (PMC13312672; doi:10.3389/fonc.2026.1820120)
Supplement: Supplementary file 1 [file DataSheet1.docx]

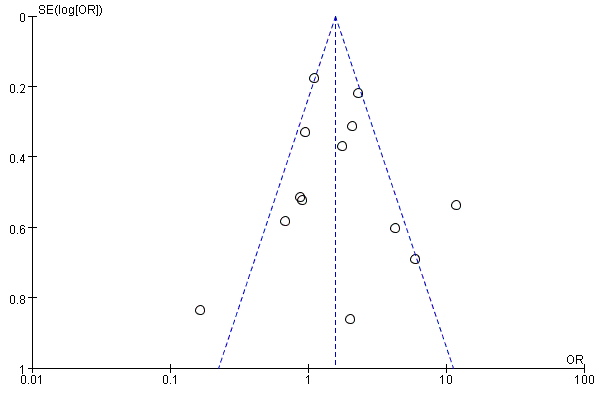


Supplementary Figure 1. Funnel plot with pseudo 95% confidence limits for the effect of angiogenesis inhibitor-based chemotherapy estimated from individual studies (horizontal axis) against the study size (vertical axis): the symmetric inverted funnel shape suggests a low likelihood of publication bias.


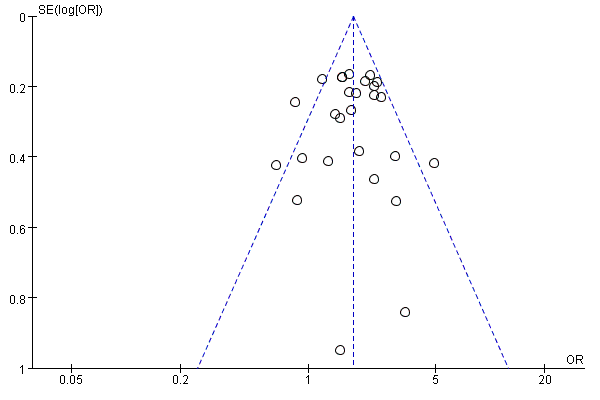


Supplementary Figure 2. Funnel plot with pseudo 95% confidence limits for the effect of angiogenesis inhibitor-based chemotherapy estimated from individual studies (horizontal axis) against the study size (vertical axis): the symmetric inverted funnel shape suggests a low likelihood of publication bias.

A


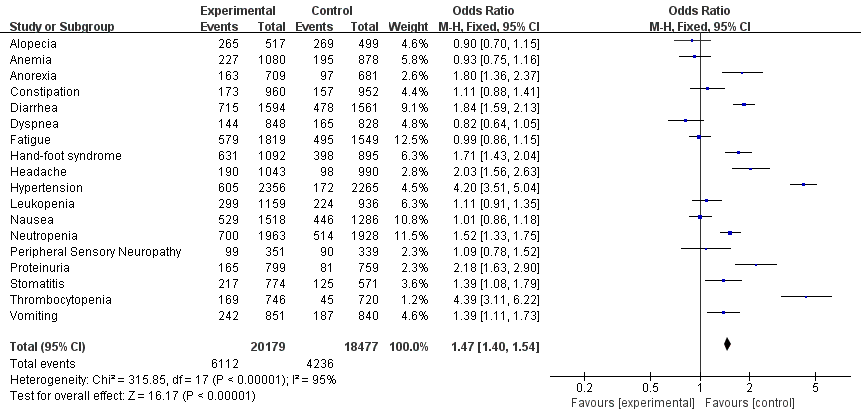


Supplementary Figure 3. Overview of safety profile. Odds ratios for adverse reactions in angiogenesis inhibitor-based versus angiogenesis inhibitor-free chemotherapy.

| **B (Anorexia)** |
| --- |


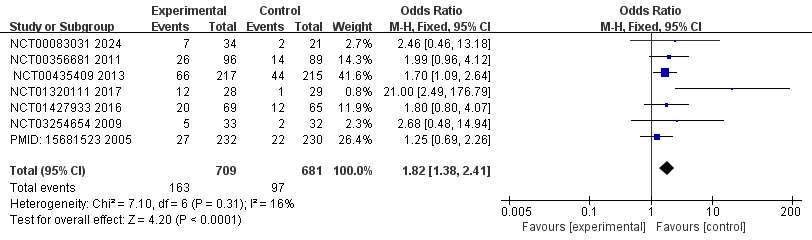


Supplementary Figure 4. Odds ratios for Anorexia mucositis in angiogenesis inhibitor-based versus angiogenesis inhibitor-free chemotherapy.

| **C (Diarrhea)** |
| --- |


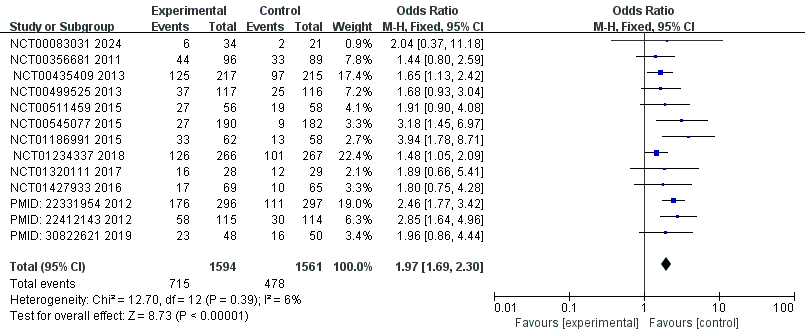


Supplementary Figure 5. Odds ratios for Diarrhea mucositis in angiogenesis inhibitor-based versus angiogenesis inhibitor-free chemotherapy.

| **D (Hand-foot syndrome)**  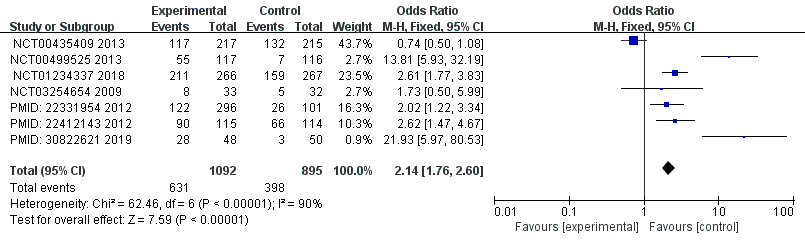 |
| --- |
| Supplementary Figure 6. Odds ratios for Hand-foot syndrome mucositis in angiogenesis inhibitor-based versus angiogenesis inhibitor-free chemotherapy. |
| **E (Headache)**  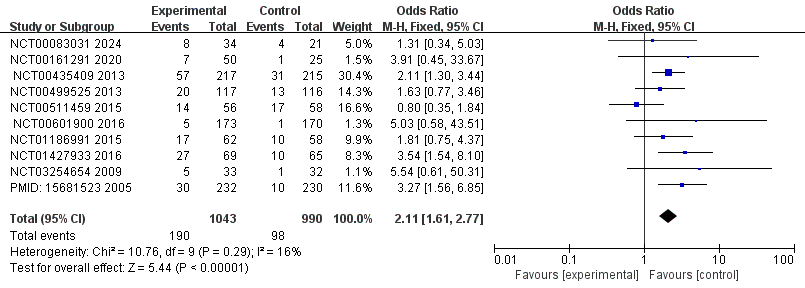  Supplementary Figure 7 .Odds ratios for Headache mucositis in angiogenesis inhibitor-based versus angiogenesis inhibitor-free chemotherapy. |
|  |
| **F (Hypertension)**  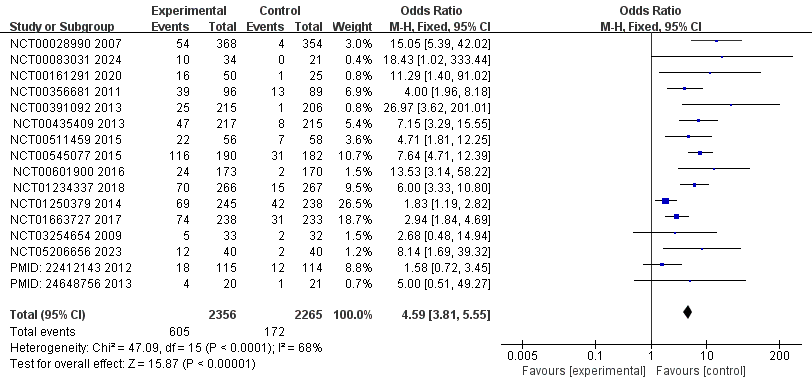 |

Supplementary Figure 8. Odds ratios for Hypertension mucositis in angiogenesis inhibitor-based versus angiogenesis inhibitor-free chemotherapy.

**G (Neutropenia)**


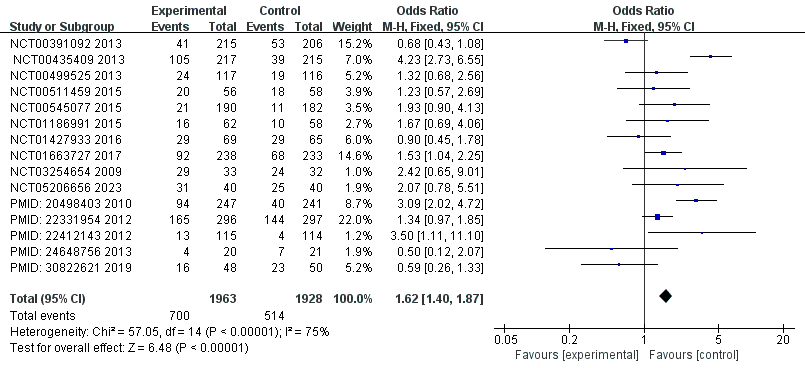


Supplementary Figure 9. Odds ratios for Neutropenia mucositis in angiogenesis inhibitor-based versus angiogenesis inhibitor-free chemotherapy.

**H (Proteinuria)**


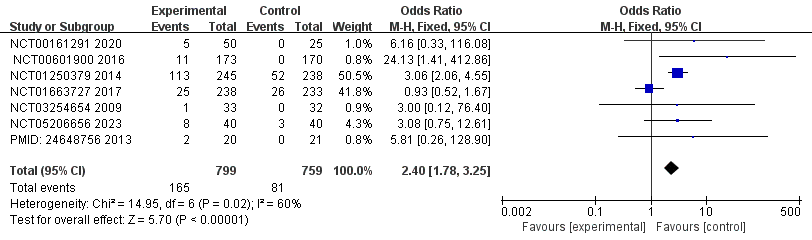


Supplementary Figure 10. Odds ratios for Proteinuria mucositis in angiogenesis inhibitor-based versus angiogenesis inhibitor-free chemotherapy.

**I (Thrombocytopenia)**


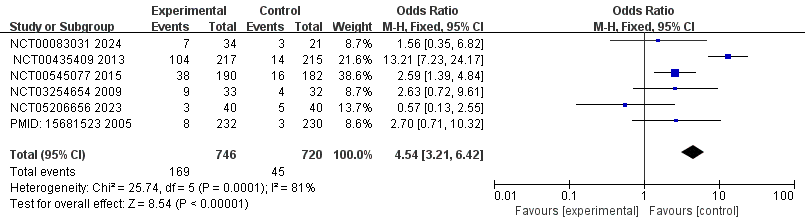


Supplementary Figure 11. Odds ratios for Thrombocytopenia mucositis in angiogenesis inhibitor-based versus angiogenesis inhibitor-free chemotherapy.
